# Photocatalytic hydrogen peroxide splitting on metal-free powders assisted by phosphoric acid as a stabilizer

**DOI:** 10.1038/s41467-020-17216-2

**Published:** 2020-07-07

**Authors:** Yasuhiro Shiraishi, Yuki Ueda, Airu Soramoto, Satoshi Hinokuma, Takayuki Hirai

**Affiliations:** 1 Research Center for Solar Energy Chemistry, and Division of Chemical Engineering, Graduate School of Engineering Science, Osaka University, Toyonaka, 560-8531 Japan; 2 Division of Materials Science and Chemistry, Faculty of Advanced Science and Technology, Kumamoto University, Kumamoto, 860-8555 Japan

**Keywords:** Photocatalysis, Artificial photosynthesis, Photocatalysis

## Abstract

Hydrogen peroxide (H_2_O_2_) has received increasing attention as an energy carrier. To achieve a sustainable energy society, photocatalytic H_2_O_2_ splitting (H_2_O_2_ (l) → H_2_ (g)^ + ^O_2_ (g); Δ*G*° = + 131 kJ mol^−1^) is a desirable reaction for on-site H_2_ generation. However, this reaction has not been reported because conventional photocatalysis decomposes H_2_O_2_ by disproportionation (H_2_O_2_ (l) → H_2_O (l) + 1/2O_2_ (g); Δ*G*° = −117 kJ mol^−1^) and by promoting H_2_O_2_ reduction instead of H^+^ reduction. Here we report the successful example of H_2_O_2_ splitting. Visible light irradiation of a graphitic carbon nitride loaded with graphene quantum dots as co-catalysts (GQDs/g-C_3_N_4_) in a H_2_O_2_ solution containing phosphoric acid (H_3_PO_4_) produces H_2_. H_3_PO_4_ associates with H_2_O_2_ via hydrogen bonding, and this stabilization of H_2_O_2_ suppresses its reduction, thus promoting H^+^ reduction. The all-organic photosystem with H_3_PO_4_ as a stabilizer may provide a basis of photocatalytic H_2_O_2_ splitting.

## Abstract (web summary)

While H_2_ can serve as a renewable fuel, its large scale production, storage, and transport are challenging. Here, authors show H_2_O_2_ to serve as a potential energy carrier via the photocatalytic production of H_2_ from stabilized H_2_O_2_ solutions and metal-free catalysts.

## Introduction

Artificial photosynthesis, which transforms earth-abundant resources into fuels by sunlight, is an urgent and challenging issue for realizing a sustainable energy society^1,2^. In the last 50 years, photocatalytic water splitting for hydrogen (H_2_) generation under sunlight irradiation (Eq. (1)) has been extensively studied for this purpose^3–5^. However, H_2_ gas has a low volumetric energy density and needs to be converted into a storable and transportable liquid energy carrier such as an organic hydride^6^ and ammonia^7^. Therefore, identifying a new artificial photosynthesis system that directly generates a liquid energy carrier is a challenge. Recently, hydrogen peroxide (H_2_O_2_) has received increasing attention as a new liquid energy carrier because it is storable and transportable and generates electricity in a direct peroxide–peroxide fuel cell (DPPFC), although careful handling is necessary owing to its property of being decomposed in the presence of some metal impurities or under heating conditions^8^. The most attractive feature is that H_2_O_2_ can be generated from earth-abundant water and oxygen (O_2_) via photocatalysis^9–11^. The photogenerated valence band holes (VB h^+^) oxidize water (O_2_ generation) and the conduction band electrons (CB e^−^) reduce O_2_ (H_2_O_2_ generation). These reactions generate H_2_O_2_ by sunlight irradiation under ambient conditions with a positive Gibbs free energy change (Eq. (2)). Therefore, photocatalytic H_2_O_2_ generation is a new potential candidate for artificial photosynthesis. Recently, we reported that the resorcinol–formaldehyde resins prepared by the high-temperature hydrothermal synthesis could successfully catalyze the above-mentioned reactions on absorbing visible light up to 620 nm^12^. Further, these metal-free resins stably produced H_2_O_2_ with 0.5% of solar-to-chemical conversion efficiency, which is comparable to the highest efficiency for photocatalytic water splitting (Eq. (1)) by metal-based powder photocatalysts^13^. Therefore, H_2_O_2_ is a new promising liquid energy carrier candidate.

1 $${\mathrm{H}}_2{\mathrm{O}}\left( {\mathrm{l}} \right)\mathop{\longrightarrow}\limits^{{hv}}{\mathrm{H}}_2\left( {\mathrm{g}} \right) + 1/2{\mathrm{O}}_2\left( {\mathrm{g}} \right)\left( {\Delta G^\circ = + 237\,{\mathrm{kJ}}\,{\mathrm{mol}}^{-1}} \right)$$

2 $${\mathrm{H}}_2{\mathrm{O}}\left( {\mathrm{l}} \right) + 1/2{\mathrm{O}}_2\left( {\mathrm{g}} \right)\mathop{\longrightarrow}\limits^{{hv}}{\mathrm{H}}_2{\mathrm{O}}_2\left( {\mathrm{l}} \right)\left( {\Delta G^\circ = + 117\,{\mathrm{kJ}}\,{\mathrm{mol}}^{-1}} \right)$$

To realize a sustainable energy society with H_2_O_2_, on-site generation of H_2_ from H_2_O_2_ solution is necessary also for its use as a hydrogen carrier. Theoretically, semiconductor photocatalysis can promote the overall H_2_O_2_ splitting. The photogenerated VB h^+^ oxidize H_2_O_2_ to produce O_2_ (Eq. (3)), and the CB e^−^ reduce H^+^ to form H_2_ (Eq. (4)). These redox reactions lead to the generation of H_2_ from H_2_O_2_ under sunlight at ambient temperature (Eq. (5)). Owing to the relatively large Gibbs free energy gain (Δ*G*° = +131 kJ mol^−1^), H_2_O_2_ splitting is potentially a new type of artificial photosynthesis reaction. Despite these advantages, photocatalytic H_2_O_2_ splitting is not reported. This is because H_2_O_2_ readily decomposes into water and O_2_ over conventional H_2_ generation photocatalysts even under dark conditions by disproportionation (Eq. (6)) on the surfaces of metal-oxide semiconductors (such as TiO_2_) or of metal particle co-catalysts (such as Pt)^14–16^. Other reasons are that H_2_O_2_ is decomposed into hydroxyl radicals on ultraviolet (UV) light absorption at *λ* < 400 nm (Eq. (7))^17,18^, and CB e^−^ reduce H_2_O_2_ (Eqs. (8) and (9)) more efficiently than H^+^ (Eq. (4)), owing to their low-lying reduction potentials^19^. The design of visible-light-driven metal-free photocatalytic systems that can selectively reduce H^+^ while suppressing H_2_O_2_ reduction is necessary.

3 $${\mathrm{H}}_2{\mathrm{O}}_2\left( {\mathrm{l}} \right) + 2{\mathrm{h}}^ + \to {\mathrm{O}}_2\left( {\mathrm{g}} \right) + 2{\mathrm{H}}^ + \left( {\mathrm{aq}} \right)\left( { + 0.68{\mathrm{V}}\,{\mathrm{vs}}\,{\mathrm{NHE}}} \right)$$

4 $$2{\mathrm{H}}^ + \left( {\mathrm{aq}} \right) + 2{\mathrm{e}}^- \to {\mathrm{H}}_2\left( {\mathrm{g}} \right)\left( {0{\mathrm{V}}\,{\mathrm{vs}}\,{\mathrm{NHE}}} \right)$$

5 $${\mathrm{H}}_2{\mathrm{O}}_2\left( {\mathrm{l}} \right)\mathop{\longrightarrow}\limits^{{hv}}{\mathrm{H}}_2\left( {\mathrm{g}} \right) + {\mathrm{O}}_2\left( {\mathrm{g}} \right)\left( {\Delta G^\circ = + 131\,{\mathrm{kJ}}\,{\mathrm{mol}}^{-1}} \right)$$

6 $${\mathrm{H}}_2{\mathrm{O}}_2\left( {\mathrm{l}} \right) \to {\mathrm{H}}_2{\mathrm{O}}\left( {\mathrm{l}} \right) + 1/2{\mathrm{O}}_2\left( {\mathrm{g}} \right)\left( {\Delta G^\circ = -117\,{\mathrm{kJ}}\,{\mathrm{mol}}^{-1}} \right)$$

7 $${\mathrm{H}}_2{\mathrm{O}}_2\left( {\mathrm{l}} \right)\mathop{\longrightarrow}\limits^{{hv\left( {\lambda < 400\,{\mathrm{nm}}} \right)}}{\mathrm{2}} \cdot{\mathrm{OH}}\left( {\mathrm{aq}} \right)$$

8 $${\mathrm{H}}_2{\mathrm{O}}_2\left( {\mathrm{l}} \right) + {\mathrm{H}}^ + \left( {\mathrm{aq}} \right) + {\mathrm{ e}}^- \to {\mathrm{H}}_2{\mathrm{O}}\left( {\mathrm{l}} \right) + \cdot {\mathrm{OH}}\left( {\mathrm{aq}} \right)\left( { + 1.14\,{\mathrm{V}}\,{\mathrm{vs}}\,{\mathrm{NHE}}} \right)$$

9 $${\mathrm{H}}_2{\mathrm{O}}_2\left( {\mathrm{l}} \right) + 2{\mathrm{H}}^ + \left( {\mathrm{aq}} \right) + 2{\mathrm{e}}^- \to {\mathrm{H}}_2{\mathrm{O}}\left( {\mathrm{l}} \right)\left( { + 1.76\,{\mathrm{V}}\,{\mathrm{vs}}\,{\mathrm{NHE}}} \right)$$

Herein, we report the successful example of photocatalytic H_2_O_2_ splitting. To avoid the undesirable H_2_O_2_ disproportionation (Eq. (6)), we used a graphitic carbon nitride (g-C_3_N_4_) organic semiconductor^20^. The g-C_3_N_4_ is less active for H_2_O_2_ disproportionation^14^, and its VB and CB levels (+2.00 and −0.63 V (vs NHE), respectively) are sufficient for H_2_O_2_ oxidation (Eq. (3)) and H^+^ reduction (Eq. (4)). We also used graphene quantum dots (GQDs) as co-catalysts; these are the carbon materials belonging to the graphene family and have attracted increasing attention as non-metal H_2_ evolution co-catalysts owing to their high electron conductivity and electron reservation capacity^21–24^. Visible-light irradiation of the GQD/g-C_3_N_4_ catalyst in a H_2_O_2_ solution containing phosphoric acid (H_3_PO_4_) at a relatively low temperature (~293 K) successfully produced H_2_. The addition of H_3_PO_4_, which is used as a stabilizer for commercially available H_2_O_2_ solution^25,26^, plays a pivotal role in this reaction. Raman spectroscopy and ab initio calculations revealed that H_3_PO_4_ associates with H_2_O_2_ via hydrogen bonding. The stabilization of H_2_O_2_ by H_3_PO_4_ inhibited H_2_O_2_ reduction (Eqs. (8) and (9)) and, hence, promoted H^+^ reduction (Eq. (4)), resulting in successful H_2_ generation.

## Results

### Preparation and characterization of catalyst

**Fig. 1:**
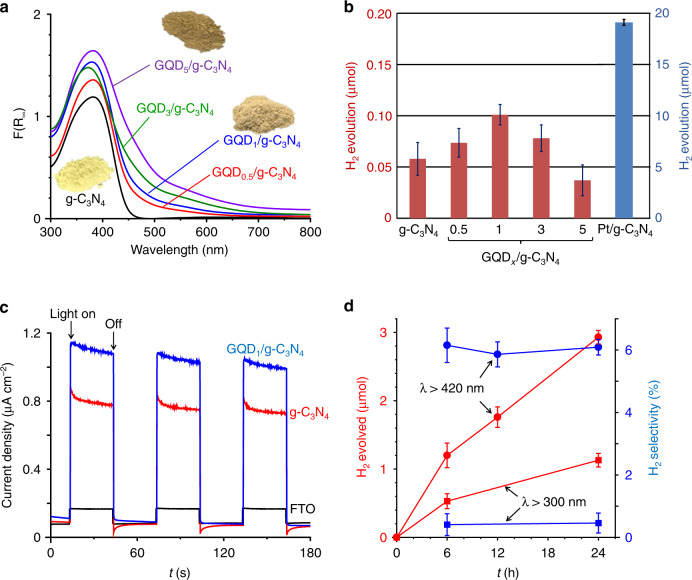
Properties of GQD_*x*_/g-C_3_N_4_ photocatalysts. **a** DR UV-vis spectra of catalysts. **b** Amounts of H_2_ evolved on the respective catalysts in water containing TEOA as a sacrificial electron donor performed in a closed system (0.1 MPa Ar). Conditions: catalyst (100 mg), a water/TEOA (9/1 v/v) mixture (30 mL), light irradiation (*λ* > 420 nm, Xe lamp), room temperature, and photoirradiation time (6 h). Error bars represent standard error (s.e.) determined by three independent experiments. **c** Photocurrent response of the catalysts measured on an FTO glass in 0.1 M Na_2_SO_4_ solution at a bias of 0.8 V vs Ag/AgCl. **d** Time-dependent change in the amount of H_2_ evolved and the H_2_ selectivity during photoirradiation of the entire wavelength light (*λ* > 300 nm) or *λ* > 420 nm light in a closed gas circulation system (3 kPa Ar). Conditions: catalyst (200 mg), H_2_O_2_ (10 mM, 100 mL), H_3_PO_4_ (1 M), temperature (293 K), and light irradiation (solar simulator with AM 1.5 G filter, 1-sun). Error bars represent standard error (s.e.) determined by three independent experiments.

**Fig. 2:**
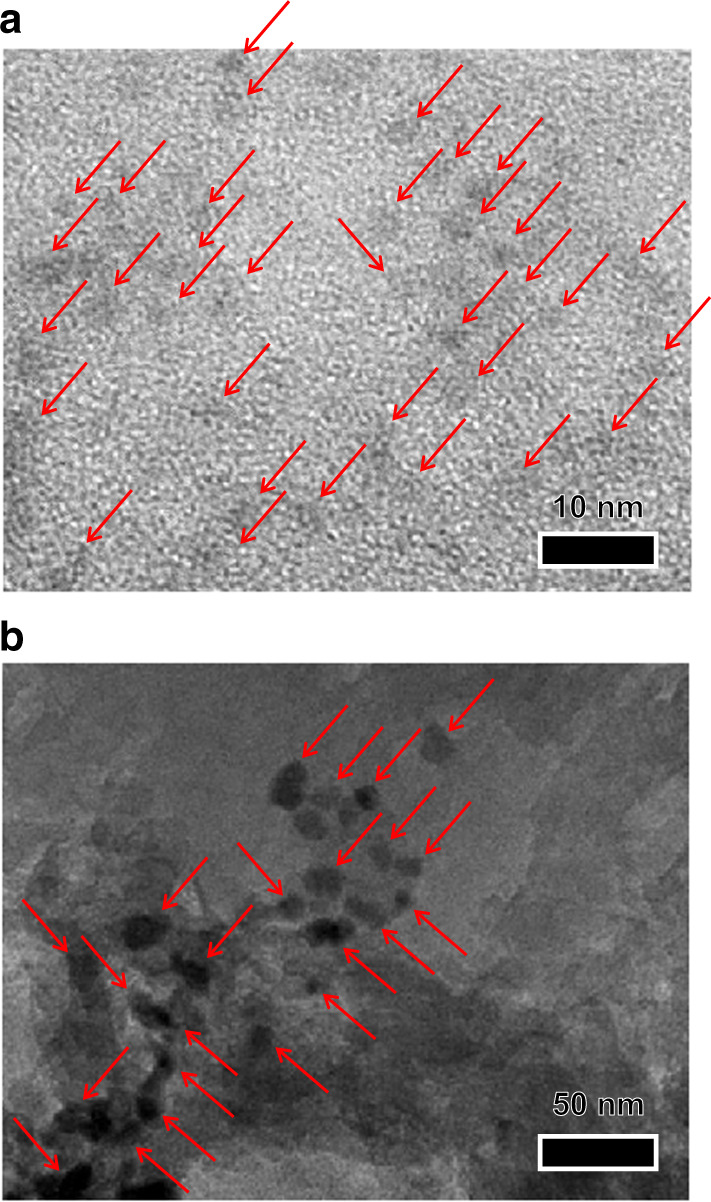
Typical TEM images. **a** GQD solution. **b** GQD_1_/g-C_3_N_4_ catalyst. The red arrows are the guide for the eyes to follow the GQD particles.

g-C_3_N_4_ powder was prepared by calcination of melamine^27^. The GQDs solution was produced by the reduction of nitrated pyrene with hydrazine followed by hydrothermal treatment^23^. The GQD_*x*_/g-C_3_N_4_ catalyst was prepared by the hydrothermal treatment of a GQDs solution containing g-C_3_N_4_, where *x* denotes the amount of GQDs-loaded relative to that of g-C_3_N_4_ [*x* (wt%) = GQDs/g-C_3_N_4_ × 100]. In the UV–visible absorption spectra of the solution (Supplementary Fig. 1), the absorption band at *λ* > 500 nm for GQDs^28^ almost disappears completely after hydrothermal treatment with g-C_3_N_4_, indicating that almost all of the GQDs in the solutions were successfully loaded on the g-C_3_N_4_ surface. In Fig. 1a, the diffuse-reflectance (DR) UV–visible spectrum of GQD_*x*_/g-C_3_N_4_ presents a band at *λ* < 470 nm, which is assigned to the bandgap transition of g-C_3_N_4_. Increasing the amount of the GQDs loaded increases the absorbance at *λ* > 500 nm, indicating that the GQDs are indeed loaded on the g-C_3_N_4_ surface. The bandgap energies of both g-C_3_N_4_ and GQD_1_/g-C_3_N_4_ were determined to be ~2.6 eV by the Tauc plot analysis (Supplementary Fig. 2), suggesting that the GQDs loading scarcely affects the band structure of g-C_3_N_4_. The powder X-ray diffraction (XRD) patterns of g-C_3_N_4_ and GQD_*x*_/g-C_3_N_4_ present peaks at 2*θ* = 27.4° (*d* = 0.325 nm), which are assigned to the (002) packing of the melem sheet (Supplementary Fig. 3)^20^, indicating that these catalysts maintain their layered stacking structure even after the GQDs loading. The scanning electron microscopy (SEM) images of both g-C_3_N_4_ and GQD_1_/g-C_3_N_4_ (Supplementary Fig. 4) indicate an amorphous solid morphology with similar sizes (~30 μm diameter). The transmission electron microscopy (TEM) results of the GQDs solution (Fig. 2, top) present dispersed GQD particles with ~3-nm diameters^23,29,30^. In contrast, the GQD_1_/g-C_3_N_4_ catalyst (Fig. 2, bottom) has larger GQD particles with ~10-nm diameters, indicating that the GQD particles are loaded onto the g-C_3_N_4_ surface via some aggregation during the hydrothermal treatment.

### Effect of GQDs as co-catalysts

The effect of the GQDs on the photocatalytic activity for H_2_ generation was studied using triethanolamine (TEOA) as a sacrificial electron donor. A TEOA/water (1/9 v/v) mixture (30 mL) containing the catalyst (100 mg) was photoirradiated for 6 h by a xenon lamp (*λ* > 420 nm) with magnetic stirring at room temperature under Ar atmosphere in a closed system (0.1 MPa). Figure 1b presents the amount of H_2_ evolved over the respective catalysts. The loading of GQDs enhances H_2_ evolution, indicating that the GQDs act as co-catalysts for H^+^ reduction. GQD_1_/g-C_3_N_4_ exhibits the highest activity; however, further GQDs loading decrease the activity. This decrease is because a larger amount of GQDs absorb more incident light (Fig. 1a) and suppresses the photoexcitation of g-C_3_N_4_. Figure 1c shows the photocurrent responses of the catalysts measured on a fluorine tin oxide (FTO) electrode. The photocurrent density of GQD_1_/g-C_3_N_4_ is higher than that of g-C_3_N_4_, indicating that the CB e^−^ photogenerated on g-C_3_N_4_ are efficiently transferred to the GQDs and electrode^31,32^. The enhanced charge separation of the h^+^ and e^−^ pairs by the GQDs loading, therefore, enhances the activity for H_2_ evolution. As shown by the blue bar in Fig. 1b, g-C_3_N_4_ when loaded with Pt particles (Pt/g-C_3_N_4_), a typical noble metal co-catalyst for H_2_ generation^33^, exhibits a much higher activity than GQD_1_/g-C_3_N_4_, where the amount of H_2_ formed is 100-fold higher than that formed on GQD_1_/g-C_3_N_4_. The activity of the GQDs as co-catalysts for H_2_ generation is lower than that of Pt.

### Photocatalytic H_2_O_2_ splitting

Photocatalytic H_2_O_2_ splitting was performed in a closed gas circulation system at a reduced pressure^34^ (3 kPa Ar) under a constant temperature. Visible light (*λ* > 420 nm) was irradiated by a solar simulator with an AM1.5 G filter (1-sun) to a H_2_O_2_ solution (1 mmol, 100 mL) containing a catalyst (200 mg) at 293 K. Table 1 lists the amounts of H_2_ and O_2_ generated and the amount of H_2_O_2_ consumed during 6 h of photoirradiation. As shown by entry 1, stirring the solution containing GQD_1_/g-C_3_N_4_ in the dark does not produce H_2_ or O_2_ and does not consume H_2_O_2_, indicating that the catalyst is indeed inactive for the disproportionation of H_2_O_2_. As shown by entry 2, photoirradiation of Pt/g-C_3_N_4_ does not produce H_2_ while decomposing almost the entire H_2_O_2_ and producing a very large amount of O_2_, suggesting that Pt significantly promote H_2_O_2_ disproportionation (Eq. (6))^14,15^. As shown by entry 3, photoirradiation of GQD_1_/g-C_3_N_4_ in pure water without H_2_O_2_ does not produce H_2_ or O_2_ because g-C_3_N_4_ is less active for water oxidation by the VB h^+^ due to its relatively negative VB level^35^. As exhibited by entry 4, photoirradiation of GQD_1_/g-C_3_N_4_ in a H_2_O_2_ solution also does not produce H_2_ while promoting O_2_ production (17 μmol) and H_2_O_2_ consumption (30 μmol), suggesting that the CB e^−^ do not reduce H^+^ (Eq. (4)) although the VB h^+^ oxidize H_2_O_2_ (Eq. (3)). This is because the CB e^−^ reduce H_2_O_2_ (Eqs. (8) and (9)) more efficiently than H^+^ reduction (Eq. (4)), owing to their low-lying reduction potentials^19^. The H_2_O_2_ reduction by CB e^−^ must therefore be suppressed to promote H^+^ reduction.

**Table 1:** Results of photocatalytic H_2_O_2_ splitting.

Entry	Temperature (K)	Catalyst	Light	Additive	H_2_ (μmol)	O_2_ (μmol)	Consumed H_2_O_2_ (μmol)
1	293	GQD_1_/g-C_3_N_4_			N.D.	N.D.	<0.1
2	293	Pt/g-C_3_N_4_	>420 nm		N.D.	125	>999^a^
3^b^	293	GQD_1_/g-C_3_N_4_	>420 nm		N.D.	N.D.	
4	293	GQD_1_/g-C_3_N_4_	>420 nm		N.D.	17.3	30
5	293	GQD_1_/g-C_3_N_4_	>420 nm	H_3_PO_4_ (0.5 M)^c^	0.4	16.9	28
6	293	GQD_1_/g-C_3_N_4_	>420 nm	H_3_PO_4_ (1 M)^d^	1.2	7.7	20
7	293	GQD_1_/g-C_3_N_4_	>420 nm	H_3_PO_4_ (2 M)^e^	0.3	4.2	13
8	293	GQD_1_/g-C_3_N_4_	>420 nm	H_2_SO_4_ (0.1 M)^d^	N.D.	15.6	25
9	293	Pt/g-C_3_N_4_	>420 nm	H_3_PO_4_ (1 M)	0.2	251	>999^a^
10	293	GQD_1_/g-C_3_N_4_	>420 nm	NaH_2_PO_4_ (1 M)^f^	0.4	49.6	78
11	303	GQD_1_/g-C_3_N_4_	>420 nm	H_3_PO_4_ (1 M)	0.5	9.4	20
12	313	GQD_1_/g-C_3_N_4_	>420 nm	H_3_PO_4_ (1 M)	N.D.	10.8	21
13	323	GQD_1_/g-C_3_N_4_	>420 nm	H_3_PO_4_ (1 M)	N.D.	8.8	25
14	293	GQD_1_/g-C_3_N_4_	>300 nm	H_3_PO_4_ (1 M)	0.5	22.1	127

Reactions were performed in a closed gas circulation system (3 kPa Ar). Conditions are: catalyst (200 mg), light source (solar simulator, 1-sun), H_2_O_2_ solution (1 mmol (10 mM), 100 mL), and photoirradiation time (6 h). All of the data are the mean values determined by the multiple experiments (at least three times) and contain ±21% deviations.

^a^Most of H_2_O_2_ decomposes while setting up the reactor owing to rapid disproportionation on the Pt particles.

^b^Pure water without H_2_O_2_ was used for the photoreaction.

^c^The pH of the solutions was ~1.2.

^d^The pH of the solution was ~1.0.

^e^The pH of the solution was ~0.6.

^f^The pH of the solution was adjusted to ~1.0 by the addition of H_2_SO_4_.

### Effect of H_3_PO_4_

It is well known that H_3_PO_4_ is generally added to commercially available H_2_O_2_ solutions as a stabilizer to inhibit the decomposition of H_2_O_2_^36,37^. Therefore, we studied the effect of H_3_PO_4_ on the photocatalytic H_2_O_2_ splitting. As exhibited by entries 5–7, visible-light irradiation of GQD_1_/g-C_3_N_4_ in a H_2_O_2_ solution containing H_3_PO_4_ produces H_2_. This is the first successful example of photocatalytic H_2_O_2_ splitting. The photocatalytic activity depends on the amount of H_3_PO_4_ added. The addition of 1 M H_3_PO_4_ (entry 6) produces the largest amount of H_2_ (1.2 μmol), but further addition (entry 7) decreases the activity. Several types of reagents such as H_3_PO_3_^37^_,_ uric acid^38^, Na_2_CO_3_^39^, KHCO_3_^40^, barbituric acid^41^, hippuric acid^42^, urea, and acetanilide^43^ have also been reported to serve as stabilizers for H_2_O_2_. However, as shown in Supplementary Table 1, the photoirradiation of GQD_1_/g-C_3_N_4_ in a H_2_O_2_ solution containing each of the respective reagents barely produces H_2_, indicating that H_3_PO_4_ is specifically effective for photocatalytic H_2_O_2_ splitting. The pH of 1 M H_3_PO_4_ solution is ~1.0. As presented by entry 8, the photoreaction, when performed in a H_2_O_2_ solution with H_2_SO_4_ (0.1 M, pH ~1), does not produce H_2_. This indicates that a low pH is not a critical factor for enhanced H_2_ generation with H_3_PO_4_. As exhibited by entry 7, addition of more H_3_PO_4_ (2 M) decreases the amount of H_2_ generated. In this case, the amounts of O_2_ generated and H_2_O_2_ consumed also decrease because the H^+^ concentration increased by a larger amount of H_3_PO_4_ (pH ~0.7) suppresses the H_2_O_2_ oxidation by the VB h^+^ (Eq. (3)). These data imply that H_3_PO_4_ may interact with H_2_O_2_ and inhibit its reduction (Eqs. (8) and (9)), resulting in the promotion of H^+^ reduction (Eq. (4)). As shown by entry 9, the Pt/g-C_3_N_4_ catalyst, when photoirradiated with 1 M H_3_PO_4_, produces H_2_; however, in the absence of H_3_PO_4_, H_2_ is not produced (entry 2). This confirms that H_3_PO_4_ interacts with H_2_O_2_ and inhibits the H_2_O_2_ reduction by the CB e^−^; however, the amount of H_2_ formed (0.2 μmol) is much smaller than that formed over GQD_1_/g-C_3_N_4_ (1.2 μmol, entry 6). In this case, almost the entire H_2_O_2_ (1 mmol) is decomposed, similar to the case without H_3_PO_4_ (entry 2), indicating that Pt particles inevitably promote H_2_O_2_ disproportionation even in the presence of H_3_PO_4_ (Eq. (6)). Therefore, the use of the all-organic GQD/g-C_3_N_4_ catalyst, which is inactive for H_2_O_2_ disproportionation, together with H_3_PO_4_, which suppresses H_2_O_2_ reduction by the CB e^−^, is effective for photocatalytic H_2_O_2_ splitting.

### Stabilization of H_2_O_2_ by H_3_PO_4_

**Fig. 3:**
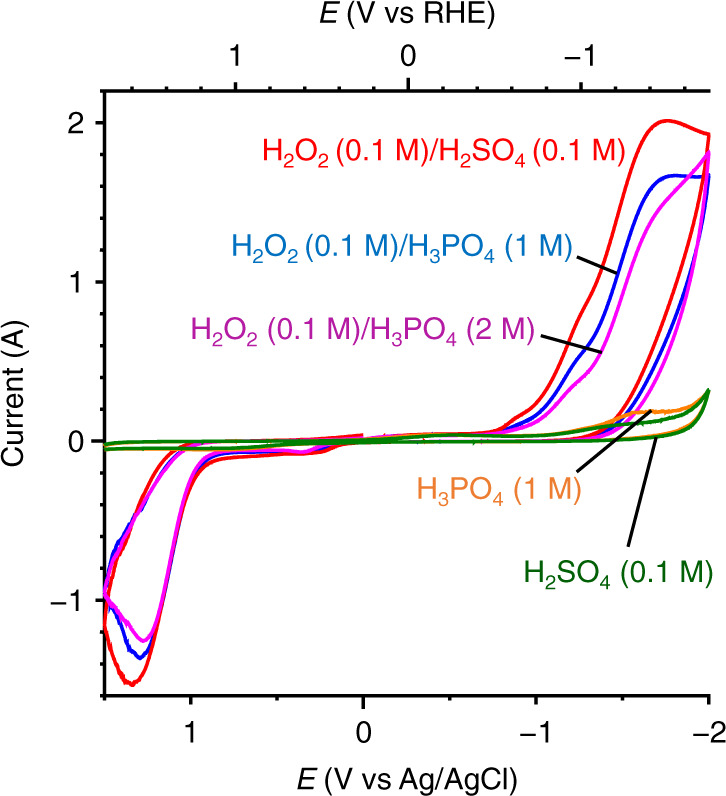
CV charts measured on a GQDs-loaded grassy carbon electrode in the different solutions. The pH of the solutions is ~1, and all the measurements were carried out under N_2_ atmosphere with 0.1 M Na_2_SO_4_ and at a scan rate of 0.1 V s^−1^.

H_3_PO_4_ is used as a stabilizer to suppress the disproportionation of H_2_O_2_ caused by metal cation impurities^44,45^, although the mechanism for their association has not been clarified yet^46–48^. The interaction between H_2_O_2_ and H_3_PO_4_ was confirmed by cyclic voltammetry (CV) measurements. Figure 3 presents the CV curves obtained with a GQDs-loaded glassy carbon electrode in a solution containing 0.1 M Na_2_SO_4_ as an electrolyte. In 0.1 M H_2_SO_4_ (pH ~1.0), as denoted by a green line, the CV curve presents a weak cathodic current at ~ –1.5 V (vs RHE), which is assigned to H^+^ reduction, where no anodic current is observed. The H_3_PO_4_ solution (1 M, pH ~1.0) presents a similar CV curve (yellow line). Addition of H_2_O_2_ (0.1 M) to the H_2_SO_4_ solution (red line) exhibits a strong cathodic current at <–0.8 V, which is assigned to H_2_O_2_ reduction (Eqs. (8) and (9))^49^, whereas a strong anodic current is observed at >1.2 V, which is assigned to H_2_O_2_ oxidation (Eq. (3)). In contrast, as shown by the blue and purple lines, addition of H_2_O_2_ to the H_3_PO_4_ solution presents cathodic current, which is weaker than that obtained in the H_2_O_2_/H_2_SO_4_ mixture (red line). These data indicate that H_2_O_2_ interacts with H_3_PO_4_ and suppresses its reduction. As reported^50^, at this pH (~1), phosphoric acid exists as a non-dissociated neutral H_3_PO_4_ form, where its mole ratio is ~94% and a mole ratio of mono-deprotonated H_2_PO_4_^−^ form is ~6%, suggesting that H_2_O_2_ interacts with the H_3_PO_4_ form.

### Raman spectroscopy

**Fig. 4:**
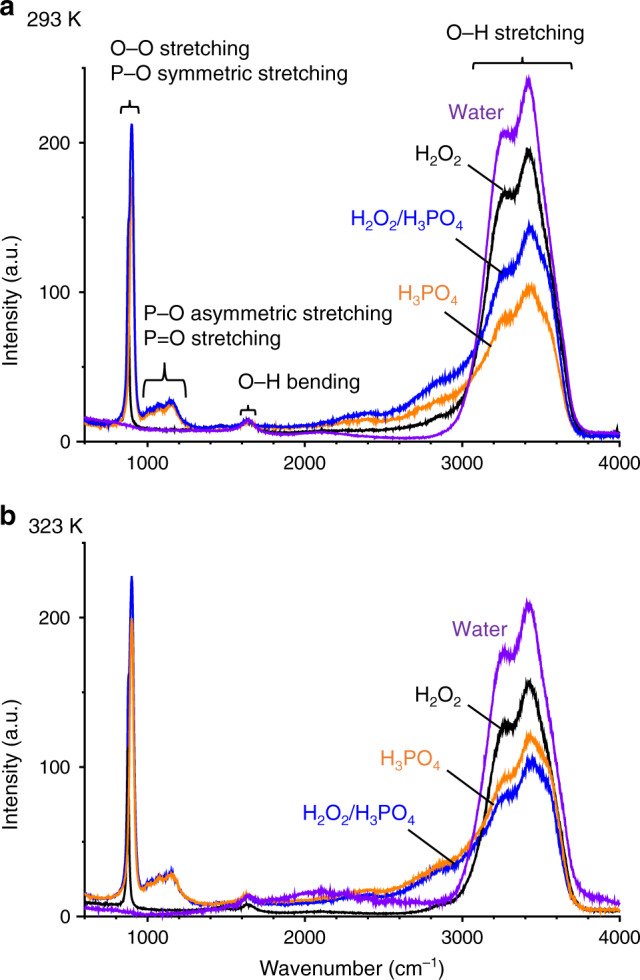
Raman spectra (600–4000 cm^−1^ region) of the respective solutions. **a** 293 K.** b** 323 K. The H_2_O_2_ and H_3_PO_4_ concentrations are 2 M and 4 M, respectively.

**Fig. 5:**
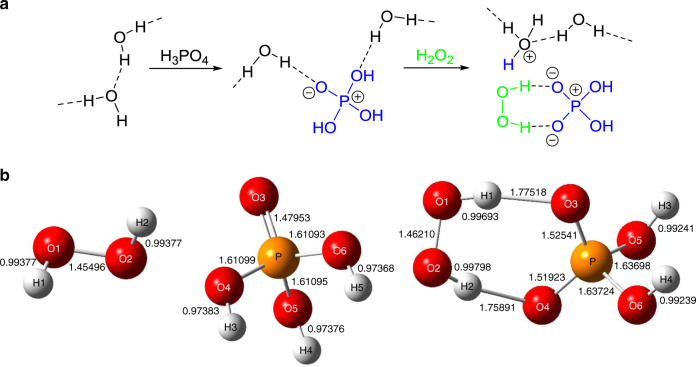
Schematic representation of H-bonding interaction and optimized structures of compounds. **a** Sequential change in H-bonding interaction between water, H_2_PO_4_^−^, and H_2_O_2_. **b** optimized structures of H_2_O_2_, H_2_PO_4_^−^, and H_2_O_2_–H_2_PO_4_^−^ complex (DFT/B3LYP/6–31 G*(d), PCM: water).

The interaction between H_2_O_2_ and H_3_PO_4_ was further studied via Raman spectroscopy at 293 K (Fig. 4a). Pure water (purple) exhibits a strong band at 2800–3800 cm^−1^ for the O–H stretching vibration of water, indicative of strong hydrogen (H-) bonding between the water molecules, as is schematically shown in Fig. 5a (left). The H_2_O_2_ solution (black) exhibits a weaker O–H band because the interaction between H_2_O_2_ and water weakens the H-bonding between the water molecules. The H_3_PO_4_ solution (orange) exhibits a much weaker O–H band because, as shown in Fig. 5a (middle), H_3_PO_4_ strongly interacts with water^50^ and significantly weakens the H-bonding between the water molecules. The addition of H_2_O_2_ to the H_3_PO_4_ solution (blue), however, increases the O–H band. This indicates that, as shown in Fig. 5a (right), H_3_PO_4_ interacts with H_2_O_2_ more strongly than with water owing to the H-bonding interaction to form the H_2_O_2_–H_2_PO_4_^−^ bidentate complex with a structure similar to the urea–H_2_PO_4_^−^ complex^51^. This may suppress the water–H_3_PO_4_ interaction and regenerate strong H-bonding interaction between the water molecules. These data suggest that H_3_PO_4_ associates with H_2_O_2_ via H-bonding to form a stabilized complex (Fig. 5a (right)), which may inhibit the H_2_O_2_ reduction by the CB e^−^.

**Fig. 6:**
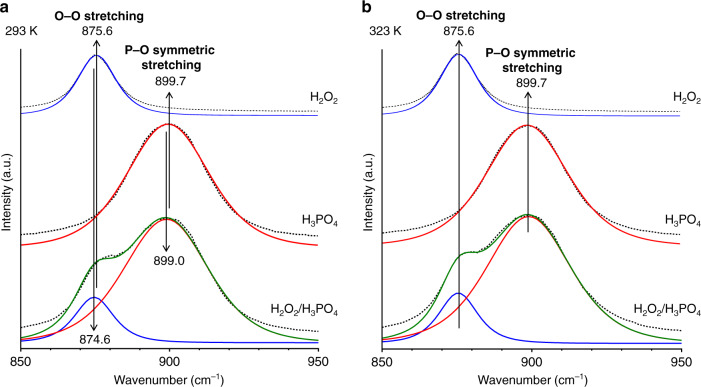
Raman spectra (850–950 cm^−1^) of (top) H_2_O_2_, (middle) H_3_PO_4_, and (bottom) a mixture of H_2_O_2_ and H_3_PO_4_ measured at different temperatures. **a** 293 K. **b** 323 K. The H_2_O_2_ and H_3_PO_4_ concentrations are 2 M and 4 M, respectively. The dots are the experimental data, and the solid lines are the deconvoluted components, where the green lines are the sum of the components.

Figure 6a presents the Raman spectroscopy results of the respective solutions in the 850–950 cm^−1^ region measured at 293 K. The H_2_O_2_ solution (top) exhibits a band at 875.6 cm^−1^, which is assigned to the O–O stretching vibration of H_2_O_2_^52^. The H_3_PO_4_ solution (middle) exhibits a band at 899.0 cm^−1^ assigned to the P–O symmetric stretching vibration of H_3_PO_4_^53^. The spectrum of the H_2_O_2_/H_3_PO_4_ mixture (bottom) can be deconvoluted into O–O and P–O stretching components. The O–O band of the mixture appears at 874.6 cm^−1^, which lies at a lower wavenumber (Δ$\widetilde v$ = −1.0 cm^−1^) than that of the band of pure H_2_O_2_ (875.6 cm^−1^). In addition, the P–O band of the mixture appears at 899.0 cm^−1^, which also lies at a lower wavenumber (Δ$\widetilde v$ = −0.7 cm^−1^) than that of pure H_3_PO_4_ (899.7 cm^−1^). These data suggest that the lengths of the O–O and P–O bonds are extended by the H-bonding between H_2_O_2_ and H_3_PO_4_ (Fig. 5a (right)).

### Ab initio calculations

To obtain further information on the interaction between H_2_O_2_ and H_3_PO_4_, the structure of the H_2_O_2_–H_2_PO_4_^−^ complex (Fig. 5a (right)) was calculated by density functional theory (DFT) using the polarizable continuum model (PCM) with water as the solvent (Supplementary Tables 3–6)^54^. Figure 5b presents the optimized structures of H_2_O_2_, H_3_PO_4_, and the H_2_O_2_–H_2_PO_4_^−^ complex, and the lengths of the respective bonds. The O–O bond length in the complex (1.462 Å) is longer than that in H_2_O_2_ (1.455 Å). In addition, the lengths of the two P–O bonds in the complex (1.525 Å (P–O3) and 1.519 Å (P–O4)) are longer than those in H_3_PO_4_ (1.480 Å (P–O3)), where the average P–O lengths of the complex (1.580 Å) is also longer than those of H_3_PO_4_ (1.578 Å). The shortened lengths of the O–O bond (Δ = 0.007 Å) and P–O bonds (Δ = 0.002 Å) by the H-bonding interaction agree reasonably with the lower wavenumber shift of the O–O and P–O stretching vibrations, as confirmed via Raman analysis (Fig. 6a). The DFT frequency calculations (Supplementary Table 2) reveal that the wavenumbers of the O–O stretching in H_2_O_2_ and the complex are 947.7 cm^−1^ and 942.8 cm^−1^^55^, respectively (Δ$\widetilde v$ = −4.9 cm^−1^), and the wavenumbers of the P–O symmetric stretching in H_3_PO_4_ and the complex are 1075 cm^−1^ and 1039 cm^−1^^56^, respectively (Δ$\widetilde v$ = −36 cm^−1^). Although the frequencies calculated by DFT are overestimated owing to the large anharmonicity of the high-frequency modes^57,58^, the lower wavenumber shifts of the O–O and P–O bonds caused by the H-bonding interaction between H_2_O_2_ and H_3_PO_4_ are consistent with the Raman data (Fig. 6a). In addition, the two O–H‧‧‧O distances of the complex are 2.749 Å (O1–H1‧‧‧O3) and 2.733 Å (O2–H2‧‧‧O4), indicative of strong and mostly electrostatic H-bonding interaction between H_2_O_2_ and H_3_PO_4_^59^. Supplementary Fig. 5 summarizes the energy diagram for the frontier molecular orbitals^60^ of the calculated models. The interaction of the highest-occupied molecular orbital (HOMO) of H_2_PO_4_^−^ with the lowest-unoccupied molecular orbital (LUMO) of H_2_O_2_ creates a frontier orbital of the complex (LUMO + 1) at an energy level higher than that of free H_2_O_2_, meaning that the H_2_O_2_ moiety of the complex is stabilized against its reduction upon external electron injection^61^. This is because the electron donation from H_2_PO_4_^−^ to H_2_O_2_ leads to a decrease in the electron affinity of the H_2_O_2_ moiety and stabilizes its orbital. The result is consistent with the suppressed H_2_O_2_ reduction (Fig. 3) and the enhanced H_2_ evolution (Table 1, entry 6) by the addition of H_3_PO_4_. Therefore, H_3_PO_4_ associates with H_2_O_2_ via H-bonding and produce a stabilized complex (Fig. 5a (right) and Fig. 5b (right)). This interaction decreases the electron affinity of H_2_O_2_ and suppresses the H_2_O_2_ reduction by the CB e^−^ (Eqs. (8) and (9)), thus resulting in the promotion of H^+^ reduction (Eq. (4)). It must be noted that addition of H_3_PO_4_ is necessary for efficient stabilization of H_2_O_2_; addition of phosphate salts is ineffective. As shown by entry 10 (Table 1), a NaH_2_PO_4_ (1 M) solution (pH ~1), when used for photoreaction, produces a lower amount of H_2_ than a H_3_PO_4_ (1 M) solution (entry 6) although fully protonated H_3_PO_4_ species exist in both solutions. Alkaline metal cations interact with H_2_O_2_ in solution^62^. The H_2_O_2_–Na^+^ interaction may weaken the H_2_O_2_–H_2_PO_4_^−^ complex, probably resulting in the decreased H_2_ evolution activity.

### Temperature effect

The stability of the H_2_O_2_–H_2_PO_4_^−^ complex depends on the temperature of the solution. As shown by entry 6 (Table 1), the photoirradiation of GQD_1_/g-C_3_N_4_ at 293 K in a H_2_O_2_ solution containing 1 M H_3_PO_4_ produces 1.2 μmol of H_2_. However, when the reaction is performed at 303 K (entry 11), the amount of H_2_ produced is decreased to 0.5 μmol, and further increase in the temperature (313 K and 323 K) does not produce H_2_ (entries 12 and 13), indicating that higher temperature is ineffective. To clarify the temperature effect on the H_2_O_2_–H_2_PO_4_^−^ interaction, Raman spectroscopy was measured at a high temperature (323 K). As displayed in Fig. 4b (orange), the O–H band of water decreases by the addition of H_3_PO_4_; this is owing to the water–H_3_PO_4_ H-bonding interaction (Fig. 5a (middle)), similar to the case at 293 K (Fig. 4a). At 293 K, the decreased O–H band increases on the addition of H_2_O_2_; this is because the formation of the H_2_O_2_–H_2_PO_4_^−^ complex regenerates the H-bonding between the water molecules. However, at 323 K, the addition of H_2_O_2_ to the H_3_PO_4_ solution barely increases the O–H band, indicating that there is no H-bonding interaction between H_2_O_2_ and H_2_PO_4_^−^. As displayed in Fig. 6a, the Raman spectrum of the H_2_O_2_/H_3_PO_4_ mixture measured at 293 K presents O–O (874.6 cm^−1^) and P–O (899.0 cm^−1^) bands, both of which lie at lower wavenumbers than those of only H_2_O_2_ (875.6 cm^−1^) and H_3_PO_4_ (899.7 cm^−1^), owing to the H_2_O_2_–H_2_PO_4_^−^ complex formation. However, at 323 K (Fig. 6b), the H_2_O_2_/H_3_PO_4_ mixture shows O–O (875.6 cm^−1^) and P–O (899.7 cm^−1^) bands at identical positions to those in H_2_O_2_ and H_3_PO_4_. These data suggest that the H_2_O_2_–H_2_PO_4_^−^ complex is destabilized at higher temperatures because a rise in temperature increases the kinetic energy of the molecules and weakens their H-bonding interaction^63^, as observed in several H-bonding systems such as dimethyl sulfoxide–water^64^ and urea–water^65^ at the similar temperature range (283–333 K). The weakened interaction at higher temperatures inevitably promotes H_2_O_2_ reduction (Eqs. (8) and (9)) and, hence, decrease H_2_ generation (Eq. (4)). The photoreaction at a relatively lower temperature is therefore necessary for efficient H_2_ generation.

### Effect of light wavelength

Visible-light irradiation is also necessary for this system. Figure 1d shows the time course for the amount of H_2_ evolved during the photocatalytic H_2_O_2_ splitting over the GQD_1_/g-C_3_N_4_ catalyst with 1 M H_3_PO_4_ at 293 K. Further, the H_2_ selectivity is defined as the ratio of the amount of H_2_ evolved to that of the consumed H_2_O_2_ (Eq. (10)). Under visible light (*λ* > 420 nm) exposure, as depicted by circle keys, the amount of H_2_ evolved increases almost linearly with time, and the H_2_ selectivity is almost constant even after prolonged photoirradiation. This indicates that the system consisting of the all-organic GQD_1_/g-C_3_N_4_ catalyst and H_3_PO_4_ as a stabilizer at a low temperature stably promotes H_2_O_2_ splitting. In addition, as presented in Supplementary Fig. 6, the recovered GQD_1_/g-C_3_N_4_ catalyst, when reused for further photoreactions, maintains the activity and the H_2_ selectivity, indicating that the catalyst is reusable without the loss of activity. However, the H_2_ selectivity is only ~6 %, indicating that the H_2_O_2_ reduction (Eqs. (8) and (9)) still occurs more efficiently than the H^+^ reduction (Eq. (4)) even in the presence of H_3_PO_4_.

10 $${\mathrm{H}}_2\,{\mathrm{selectivity}}\left( {\mathrm{\% }} \right) = \frac{{\left[ {{\mathrm{H}}_2\,{\mathrm{evolved}}\left( {{\upmu {\mathrm{mol}}}} \right)} \right]}}{{\left[ {{\mathrm{H}}_2{\mathrm{O}}_2\,{\mathrm{consumed}}\left( {{\upmu {\mathrm{mol}}}} \right)} \right]}} \times 100$$

The square keys in Fig. 1d denote the results obtained under irradiation of the entire wavelength light (*λ* > 300 nm) from the solar simulator (Supplementary Fig. 7). The amount of H_2_ evolved is much smaller than that under *λ* > 420 nm irradiation. As summarized in Table 1 (entry 14), *λ* > 300 nm irradiation for 6 h produces a larger amount of O_2_ (22 μmol) than the *λ* > 420 nm irradiation (7.7 μmol, entry 5). This indicates that UV irradiation enhances the catalyst photoexcitation and efficiently promotes H_2_O_2_ oxidation by the VB h^+^ (Eq. (3)), clearly suggesting that UV irradiation suppresses H^+^ reduction (Eq. (4)). The *λ* > 300 nm irradiation decomposes a larger amount of H_2_O_2_ (127 μmol) than the *λ* > 420 nm irradiation (20 μmol, entry 6); this is owing to the photodecomposition of H_2_O_2_ by the absorbed UV light (Eq. (7)), as confirmed by the absorption spectra of the H_2_O_2_ solution in the UV region (Supplementary Fig. 8). This suggests that the H_2_O_2_–H_2_PO_4_^−^ complex absorbs UV light and undergoes destabilization. The weakened H-bonding of the complex may therefore inevitably promote H_2_O_2_ reduction (Eqs. (8) and (9)), thus inhibiting the H^+^ reduction (Eq. (4)). As displayed in Fig. 1d (square), the H_2_ selectivity with *λ* > 300 nm irradiation is only ~0.5% owing to the accelerated H_2_O_2_ decomposition, indicating that visible-light irradiation is necessary for efficient photocatalytic H_2_O_2_ splitting. Even under visible-light irradiation, the H_2_ selectivity is ~6%; therefore, further selectivity enhancement is necessary for practical applications. Nevertheless, the successful example presented here based on the combination of an all-organic photocatalyst and H_3_PO_4_ as a stabilizer under sunlight illumination presents significant potential for photocatalytic H_2_O_2_ splitting.

## Discussion

Visible-light irradiation of the all-organic GQD/g-C_3_N_4_ catalyst in a H_2_O_2_ solution containing H_3_PO_4_ at a relatively low temperature (~293 K) could successfully promote H_2_ generation by photocatalytic H_2_O_2_ splitting. The Raman spectroscopy and DFT calculations revealed that H_3_PO_4_ associates with H_2_O_2_ via H-bonding interaction and forms a H_2_O_2_–H_2_PO_4_^−^ complex at a low temperature. This decreases the electron affinity of H_2_O_2_ and suppresses its reduction, thus promoting H^+^ reduction (H_2_ generation). The present system stably produces H_2_ even after prolonged photoirradiation, and the catalyst is reusable without the loss of activity and H_2_ selectivity. This photocatalytic system offers several advantages: a metal-free photocatalyst and inexpensive acid (H_3_PO_4_) can be used for the reaction; visible light, a main constituent of sunlight irradiation, can be used as the light source; and on-site H_2_ generation from a transportable and storable energy carrier (H_2_O_2_) can be achieved. At present, H_2_ selectivity is only ~6% relative to the amount of H_2_O_2_ consumed, and further improvement of its selectivity is necessary for practical applications. Nevertheless, the successful example presented here based on an all-organic catalyst with H_3_PO_4_ as a stabilizer may contribute to the design of a highly efficient system for the on-site photocatalytic generation of H_2_ from a H_2_O_2_ solution.

## Methods

### Catalyst preparation

The g-C_3_N_4_ powder was prepared by calcination of melamine at 823 K (heating rate: 2.3 K min^−1^, holding time: 4 h)^27^. The GQDs solution was synthesized as follows:^30^ pyrene (2 g) was stirred in concentrated HNO_3_ (160 mL) at 353 K for 12 h. Water (2 L) was added to the resultant, and the solids formed were recovered by filtration. The solids were dispersed in hydrazine hydrate solution (1.0 M, 40 mL), ultrasonicated for 2 h, and left in an autoclave at 423 K for 10 h. Filtration of the resultant by a microporous membrane afforded a 0.5-g L^−1^ GQDs solution. The GQD_*x*_/g-C_3_N_4_ catalysts [*x* (wt%) = 0.5, 1.0, 3.0, and 5.0] were prepared as follows: g-C_3_N_4_ (0.4 g) was added to 40 mL of the GQDs solution (0.05, 0.1, 0.3, and 0.5 g L^−1^) and stirred for 1 h at 298 K. The mixture was left in an autoclave at 423 K for 4 h. The solids were recovered by centrifugation, washed with water, and dried at 353 K, affording GQD_*x*_/g-C_3_N_4_ as brown powders. A Pt/g-C_3_N_4_ catalyst (Pt loading: 0.3 wt%) was prepared as follows:^33^ g-C_3_N_4_ (1.0 g) was added to water (30 mL) containing H_2_PtCl_6_‧6H_2_O (8.0 mg) and evaporated with vigorous stirring at 373 K. The obtained powders were reduced under H_2_ flow (0.1 mL min^−1^) at 673 K (heating rate: 10 K min^−1^, holding time: 2 h).

### Photoreaction

H_2_ generation from a TEOA solution was performed in a closed system (0.1 MPa). The catalyst (100 mg) was added to a 10 vol% TEOA solution (30 mL) within a glass tube (φ 35 mm; capacity, 50 mL). The tube was sealed with a rubber cap and ultrasonicated for 5 min, and Ar gas was bubbled through the solution for 10 min. The tube was photoirradiated (*λ* > 420 nm) using a 2-kW Xe lamp (USHIO Inc.) with magnetic stirring at room temperature. After the reaction, the amount of H_2_ formed was determined by gas chromatography-thermal conductivity detector (GC-TCD) (Shimadzu; GC-8A). Photocatalytic H_2_O_2_ splitting was performed in a closed gas circulation system connected to GC-TCD^66^. The catalyst (200 mg) was added to a H_2_O_2_ solution (1 mmol, 100 mL) within a quartz cell. The cell was connected to a gas circulation system, and the system was depressurized to 3 kPa after repeated Ar purging. The cell was immersed in a temperature-controlled water bath (±0.1 K) and photoirradiated at *λ* > 420 nm using a solar simulator equipped with AM1.5 G filter (SAN-EI Electric Inc.; XES-40S3, 1-sun) with magnetic stirring^12^. The amounts of H_2_O_2_ in the solutions were determined by redox titration with KMnO_4_.

### Analysis

The electrochemical measurements were performed in a conventional three-electrode cell using an electrochemical system (ALS700E, BAS Inc.), where Ag/AgCl and Pt wire electrodes were used as the reference and counter electrodes, respectively^66^. For the photocurrent measurements, a catalyst-loaded FTO glass was used as the working electrode. Catalyst (20 mg) was added to a mixture of water (4 mL), 2-propanol (1.5 mL), and 5 wt% Nafion solution (100 μL)^67^, and ultrasonicated for 10 min. The slurry was placed onto an FTO glass and dried at room temperature. The film was formed to 0.5 cm × 0.5 cm square, where the undeposited parts were coated with an epoxy resin. For the CV measurements, a glassy carbon electrode loaded with GQD was used as the working electrode, where the GQD solution was placed on the electrode and dried at room temperature. All the measurements were performed after N_2_ bubbling through the solution for 1 h. Raman spectra were measured on a confocal Raman microscope (LabRAM HR-800, HORIBA) with a YAG laser (532 nm line) as the excitation source, where the laser power was 100 mW and the total data accumulation time was 100 s, respectively. DR UV-vis, XRD, SEM, and TEM observations were performed according to the procedures described in the literature^12^.

### Computational details

The molecular structures were optimized by the B3LYP functional (B3LYP/6-31G*(d)) using the polarizable continuum model (PCM) with water as the solvent^54^ in the Gaussian 03 program. Cartesian coordinates for the models used are summarized at the end of the Supplementary Material.

## Supplementary information

Supplementary Information

Peer Review File

## Data Availability

All experimental data within the article and its Supplementary Information are available from the corresponding author upon reasonable request.

## References

[1] Concepcion JJ, House RL, Papanikolas JM, Meyer TJ (2012). Chemical approaches to artificial photosynthesis. Proc. Natl Acad. Sci. USA.

[2] Lewis NS (2016). Developing a scalable artificial photosynthesis technology through nanomaterials by design. Nat. Nanotechnol..

[3] Chen S, Takata T, Domen K (2017). Particulate photocatalysts for overall water splitting. Nat. Rev. Mater..

[4] Hisatomi T, Domen K (2019). Reaction systems for solar hydrogen production via water splitting with particulate semiconductor photocatalysts. Nat. Catal..

[5] Kudo A, Miseki Y (2009). Heterogeneous photocatalyst materials for water splitting. Chem. Soc. Rev..

[6] Teichmann D, Arlt W, Wasserscheid P (2012). Liquid organic hydrogen carriers as an efficient vector for the transport and storage of renewable energy. Int. J. Hydrog. Energy.

[7] Guo J, Chen P (2017). Catalyst: NH_3_ as an energy carrier. Chem.

[8] Fukuzumi S (2017). Production of Liquid Solar Fuels and Their Use in fuel cells. Joule.

[9] Fukuzumi S, Lee YM, Nam W (2018). Solar-driven production of hydrogen peroxide from water and dioxygen. Chem. Eur. J..

[10] Haider Z, Cho H-I, Moon G-H, Kim H-I (2019). Minireview: selective production of hydrogen peroxide as a clean oxidant over structurally tailored carbon nitride photocatalysts. Catal. Today.

[11] Hou, H., Zeng, X., & Zhang, X. Production of hydrogen peroxide through photocatalytic processes: a critical review of recent advances. *Angew. Chem. Int. Ed*. 10.1002/anie.201911609 (2019).

[12] Shiraishi Y (2019). Resorcinol–formaldehyde resins as metal-free semiconductor photocatalysts for solar-to-hydrogen peroxide energy conversion. Nat. Mater..

[13] Qi Y (2018). Redox-based visible-light-driven z-scheme overall water splitting with apparent quantum efficiency exceeding 10%. Joule.

[14] Shiraishi Y (2014). Highly selective production of hydrogen peroxide on graphitic carbon nitride photocatalyst activated by visible light. ACS Catal..

[15] Jaouen F, Dodelet JP (2009). O_2_ reduction mechanism on non-noble metal catalysts for PEN fuel cells part I: experimental rates of O_2_ electroreduction, H_2_O_2_ electroreduction, and H_2_O_2_ disproportionation. J. Phys. Chem. C.

[16] Li Z, Kong C, Lu G (2016). Visible photocatalytic water splitting and photocatalytic two-electron oxygen formation over Cu- and Fe-doped g-C_3_N_4_. J. Phys. Chem. C.

[17] Sahel K (2016). Hydrogen peroxide and photocatalysis. Appl. Catal. B Environ..

[18] Goldstein S, Aschengrau D, Diamant Y, Rabani J (2007). Photolysis of aqueous H_2_O_2_: quantum yield and applications for polychromatic UV actinometry in photoreactors. Environ. Sci. Technol..

[19] Bockris JO’M, Oldfield LF (1955). The oxidation-reduction reactions of hydrogen peroxide at inert metal electrodes and mercury cathodes. Trans. Faraday Soc..

[20] Wang X (2009). A metal-free polymeric photocatalyst for hydrogen production from water under visible light. Nat. Mater..

[21] Wang L (2014). Gram-scale synthesis of single-crystalline graphene quantum dots with superior optical properties. Nat. Commun..

[22] Min S, Hou J, Lei Y, Ma X, Lu G (2017). Facile one-step hydrothermal synthesis toward strongly coupled TiO_2_/graphene quantum dots photocatalysts for efficient hydrogen evolution. Appl. Surf. Sci..

[23] Melo Jr,MA, Osterloh FE (2018). Defect states control effective band gap and photochemistry of graphene quantum dots. ACS Appl. Mater. Interfaces.

[24] Gao Y (2018). Graphene quantum–dot–modified hexagonal tubular carbon nitride for visible–light photocatalytic hydrogen evolution. ChemCatChem.

[25] Kim NH, Lim JH, Kim SY, Chang EG (2003). Effect of phosphoric acid stabilizer on copper and tantalum nitride. CMP.

[26] Wegner, P. C. Hydrogen peroxide stabilizer and resulting product and applications. US patent US20030151024 A1 (2003).

[27] Yan SC, Li ZS, Zou ZG (2009). Photodegradation performance of g-C_3_N_4_ fabricated by directly heating melamine. Langmuir.

[28] Yu Y, Ren J, Meng M (2013). Photocatalytic hydrogen evolution on graphene quantum dots anchored TiO_2_ nanotube-array. Int. J. Hydrog. Energy.

[29] Wang R (2017). Graphene quantum dots modified g-C_3_N_4_ for enhanced photocatalytic oxidation of ammonia performance. RSC Adv..

[30] Pan D (2015). Efficient separation of electron-hole pairs in graphene quantum dots by TiO_2_ heterojunctions for dye degradation. ACS Sustain. Chem. Eng..

[31] Zou JP (2016). Synthesis and efficient visible light photocatalytic H_2_ evolution of a metal-free g-C_3_N_4_/graphene quantum dots hybrid photocatalyst. Appl. Catal. B Environ..

[32] Liu J (2017). Graphene quantum dots modified mesoporous graphite carbon nitride with significant enhancement of photocatalytic activity. Appl. Catal. B Environ..

[33] Shiraishi Y (2014). Platinum nanoparticles strongly associated with graphitic carbon nitride as efficient co-catalysts for photocatalytic hydrogen evolution under visible light. Chem. Commun..

[34] Wang Q (2016). Scalable water splitting on particulate photocatalyst sheets with a solar-to-hydrogen energy conversion efficiency exceeding 1%. Nat. Mater..

[35] Shiraishi Y (2014). Sunlight-driven hydrogen peroxide production from water and molecular oxygen by metal-free photocatalysts. Angew. Chem. Int. Ed..

[36] Achenbach, K. Process for etching copper printed circuits. US patent US3373113A (1968).

[37] Pougherty, E. F. Stabilized hydrogen peroxide. US patent US4981662A (1991).

[38] Hooper, G. W. Catalytic production of hydrogen peroxide from its elements. US patent US3336112A (1967).

[39] Lee, H. H. B., Park, A. H. & Oloman, C. Stability of hydrogen peroxide in sodium carbonate solution. *Tappi J.* **83**, 1–9 (2000).

[40] Fuku K (2017). Photoelectrochemical hydrogen peroxide production from water on a WO_3_/BiVO_4_ photoanode and from O_2_ on an Au cathode without external bias. Chem. Asian J..

[41] Baum, G. Stabilized peroxide solution. US patent US1758920A (1930).

[42] Weber, F. W. Stable product containing hydrogen peroxide and method of making the same. US patent US1210570A (1917).

[43] Malin, M. J. & Sclafani, L. D. Stabilized aqueous hydrogen peroxide solution. US patent US4744968A. (1988).

[44] Greenspan, F. P. Method of improving stability of concentrated hydrogen peroxide in contact with stainless and aluminum alloys. US patent US2782100A (1957).

[45] Shimokawa, S., Namikawa, K. & Murakami, S. Stabilized refined aqueous hydrogen peroxide solution. Jpn. Kokai Tokkyo Koho JP07-081906A (1995).

[46] Roth Jr,EM, Shanley ES (1953). Stability of Pure Hydrogen Peroxide. Ind. Eng. Chem..

[47] Sun, H., Leonard, J. J. & Shalit, H. Hydrogen peroxide production. US patent US4393038A (1983).

[48] Shang M, Noël T, Su Y, Hessel V (2016). High pressure direct synthesis of adipic from cyclohexene and hydrogen peroxide via capillary microreactors. Ind. Eng. Chem. Res..

[49] Patra S, Munichandraiah N (2009). Electrochemical reduction of hydrogen peroxide on stainless steel. J. Chem. Sci..

[50] Śmiechowski M, Gojto E, Stangret J (2009). Systematic study of hydration patterns of phosphoric(V) Acid and its mono-, di-, and tripotassium salts in aqueous solution. J. Phys. Chem. B.

[51] Kostansek EC, Busing WR (1972). A single-crystal neutron-diffraction study of urea–phosphoric acid. Acta Crystallogr. B.

[52] Moreno T (2011). Quantitative raman determination of hydrogen peroxide using the solvent as international standard: online application in the direct synthesis of hydrogen peroxide. Chem. Eng. J..

[53] Rudolph WW (2010). Raman- and infrared-spectroscopic investigations of dilute aqueous phosphoric acid solutions. Dalton Trans..

[54] Cossi M, Barone V, Cammi R, Tomasi J (1996). Ab initio study of solvated molecules: a new implementation of the polarizable continuum model. Chem. Phys. Lett..

[55] Gonzalez L, Mo O, Yanez M (1997). High-level ab initio versus D. F. T. calculations on (H_2_O_2_)_2_ and H_2_O_2_–H_2_O complexes as prototypes of multiple hydrogen bond systems. J. Comput. Chem..

[56] Ahmed AB (2009). Crystal structure, vibrational spectra and theoretical studies of l-histidinium dihydrogen phosphate-phosphoric acid. J. Mol. Struct..

[57] EI-Azhary AA, Suter HU (1996). Comparison between optimized geometries and vibrational frequencies calculated by DFT methods. J. Phys. Chem..

[58] Dkhissi A, Adamowickz L, Maes G (2000). Density functional theory study of the hydrogen-bonded pyridine–H_2_O complex: a comparison with RHF and MP2 methods and with experimental data. J. Phys. Chem. A.

[59] Jeffrey GA (1997). An Introduction to Hydrogen Bonding..

[60] Fukui K (1982). Role of frontier orbitals in chemical reactions. Science.

[61] Heinrich N, Koch W, Frenking G (1986). On the use of Koopmans’ theorem to estimate negative electron affinities. Chem. Phys. Lett..

[62] Matheson NR, Wong PS, Schuyler M, Travis J (1981). Interaction of human α-1-proteinase inhibitor with neutrophil myeloperoxidase. Biochemistry.

[63] Dougherty RC (1998). Temperature and pressure dependence of hydrogen bond strength: a perturbation molecular orbital approach. J. Chem. Phys..

[64] Oh KI, Rajesh K, Stanton JF, Baiz CR (2017). Quantifying hydrogen-bond populations in dimethyl sulfoxide/water mixtures. Angew. Chem. Int. Ed..

[65] Mafy NN, Afrin T, Rahman MM, Mollah MYA, Susan MABH (2015). Effect of temperature perturbation on hydrogen bonding in aqueous solutions of different urea concentrations. RSC Adv..

[66] Shiraishi Y (2020). Photocatalytic dinitrogen fixation with water on bismuth oxychloride in chloride solutions for solar-to-chemical energy conversion. J. Am. Chem. Soc..

[67] Wang X, Cheng J, Yu H, Yu J (2017). A facile hydrothermal synthesis of carbon dots modified g-C_3_N_4_ for enhanced photocatalytic H_2_-evolution performance. Dalton Trans..

