# Supplementary material for: Photocatalytic hydrogen peroxide splitting on metal-free powders assisted by phosphoric acid as a stabilizer
Source: Nat Commun. 2020 Jul 7;11:3386. doi: 10.1038/s41467-020-17216-2 (PMC7341847; doi:10.1038/s41467-020-17216-2)
Supplement: Supplementary file 1 — Supplementary Information [file 41467_2020_17216_MOESM1_ESM.pdf]

Supplementary Information for

# Photocatalytic hydrogen peroxide splitting on metal-free powders assisted by phosphoric acid as a stabilizer

Shiraishi et al.

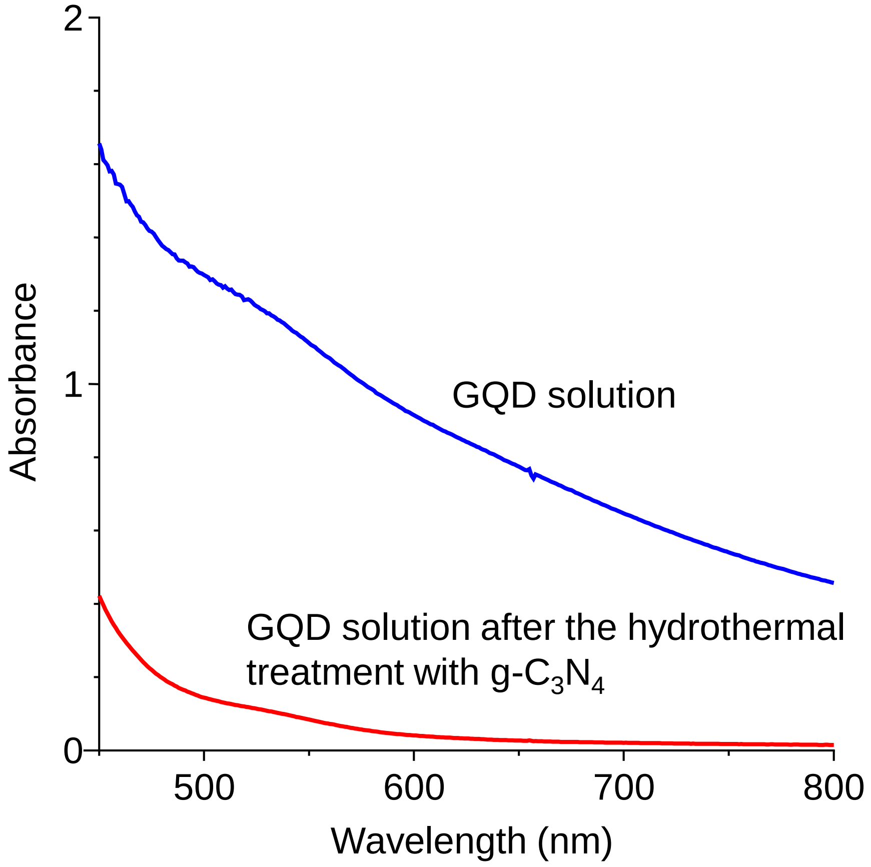


**Supplementary Fig. 1 | UV-vis absorption spectra of the GQD solutions (blue) before and (red) after hydrothermal treatment with g-$C_3N_4$.**

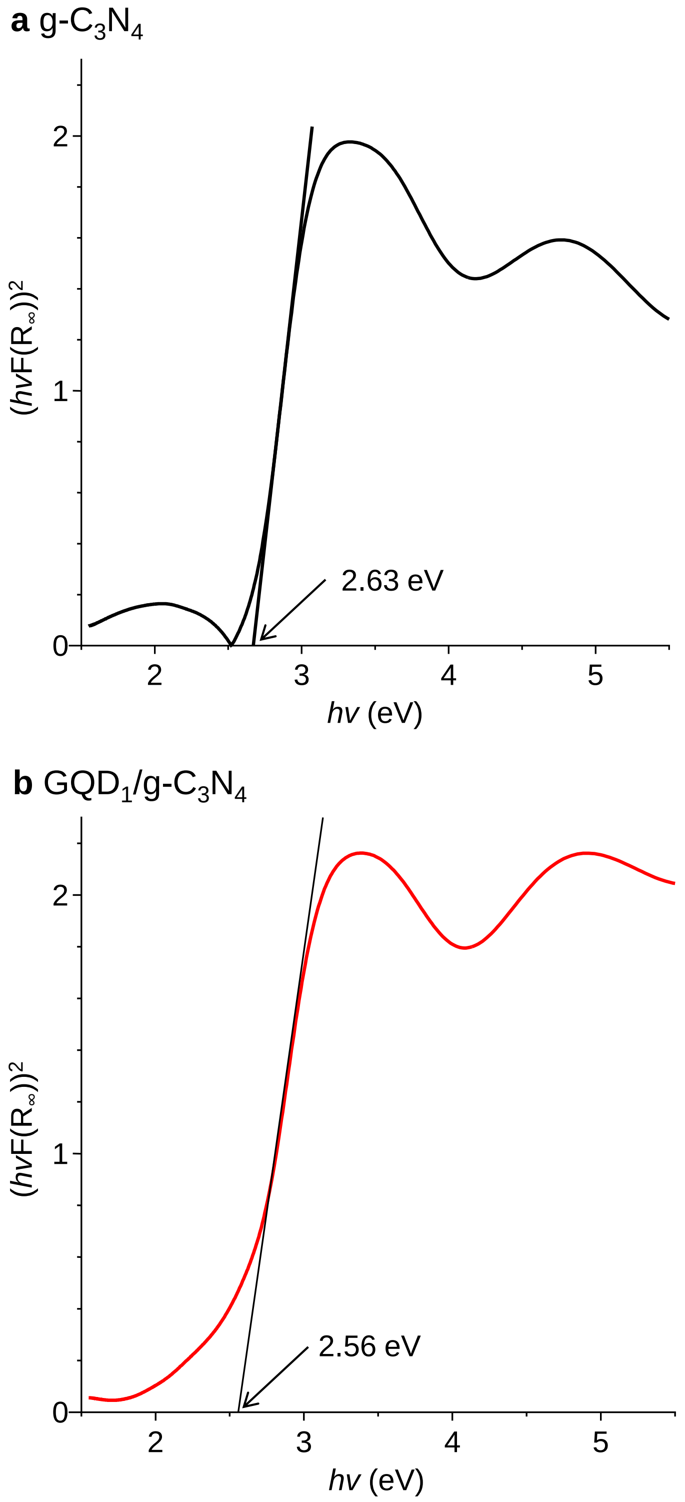


**Supplementary Fig. 2 | Tauc plots of the DR UV-vis spectra of (a) g-$C_3N_4$ and (b) $GQD_1$/g-$C_3N_4$.**

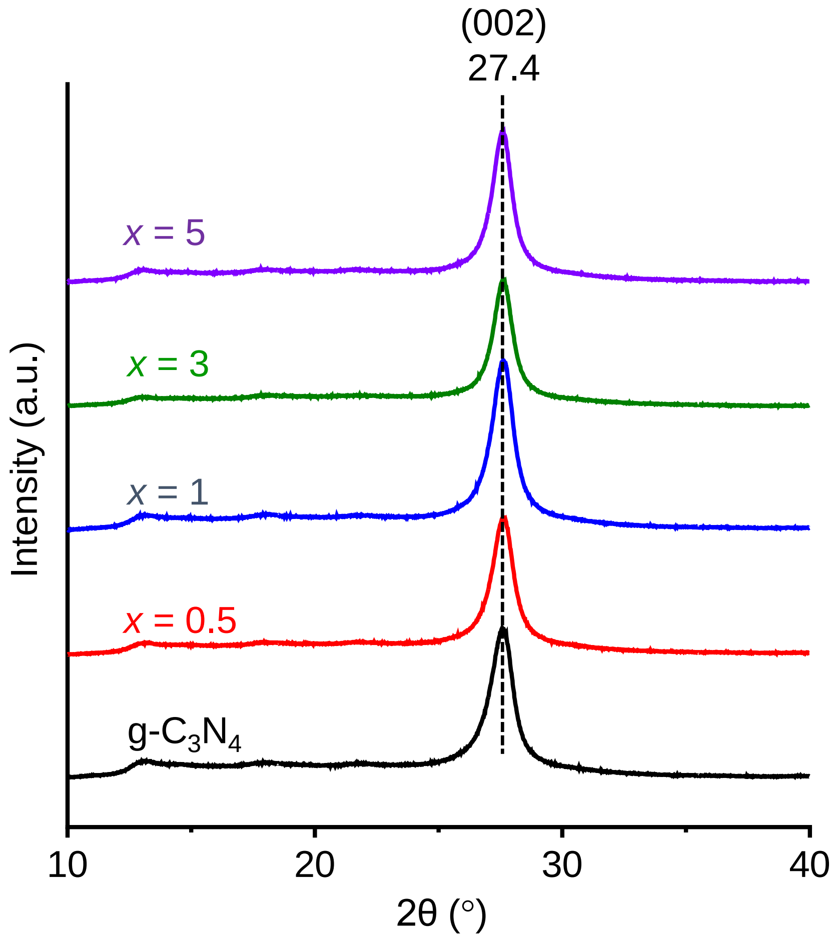


**Supplementary Fig. 3 | Powder XRD patterns of g-$C_3N_4$ and GQD$_x$/g-$C_3N_4$ catalysts.**

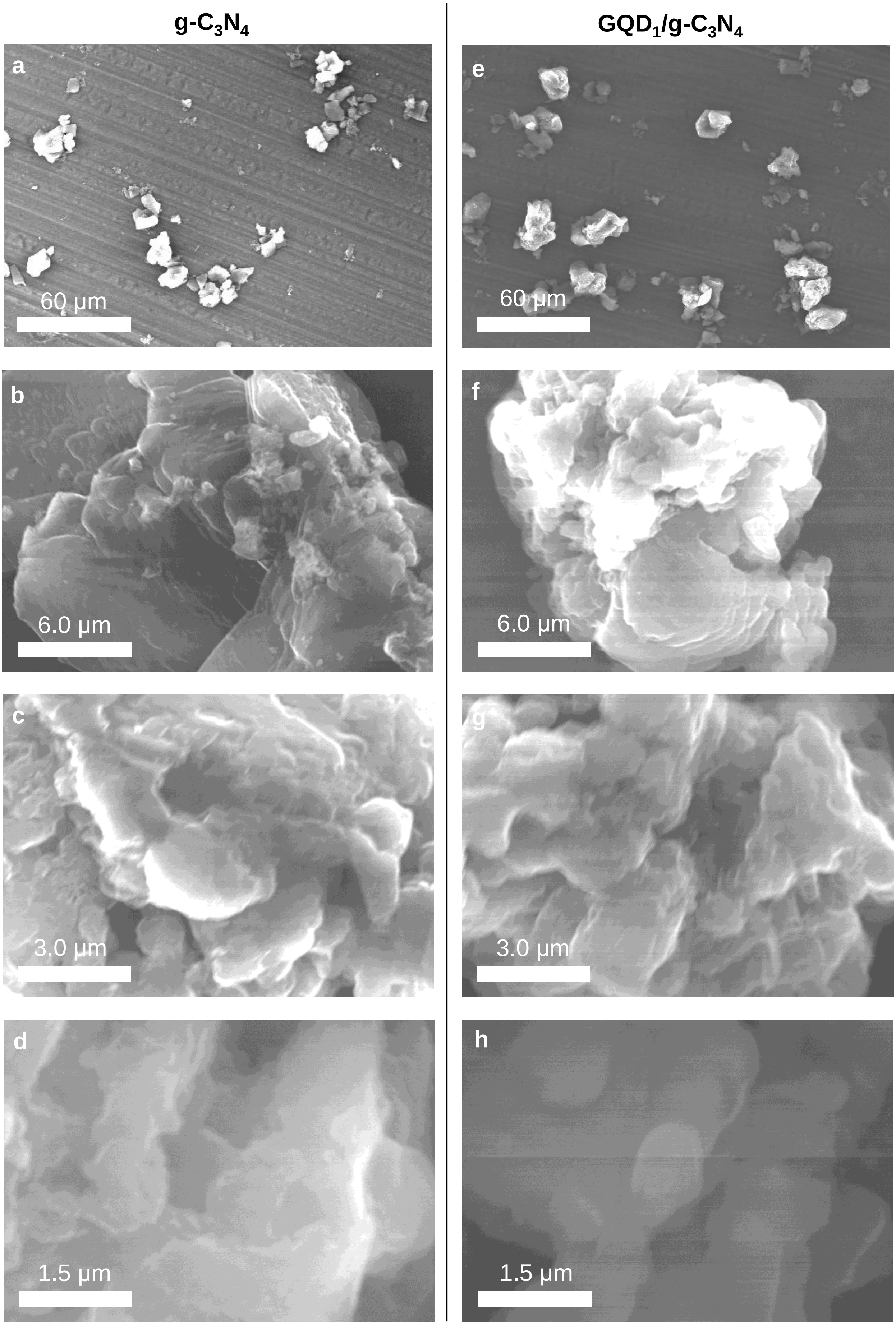


**Supplementary Fig. 4 | Typical SEM images of (a–d) g-$C_3N_4$ and (e–h) $GQD_1$/g-$C_3N_4$.**

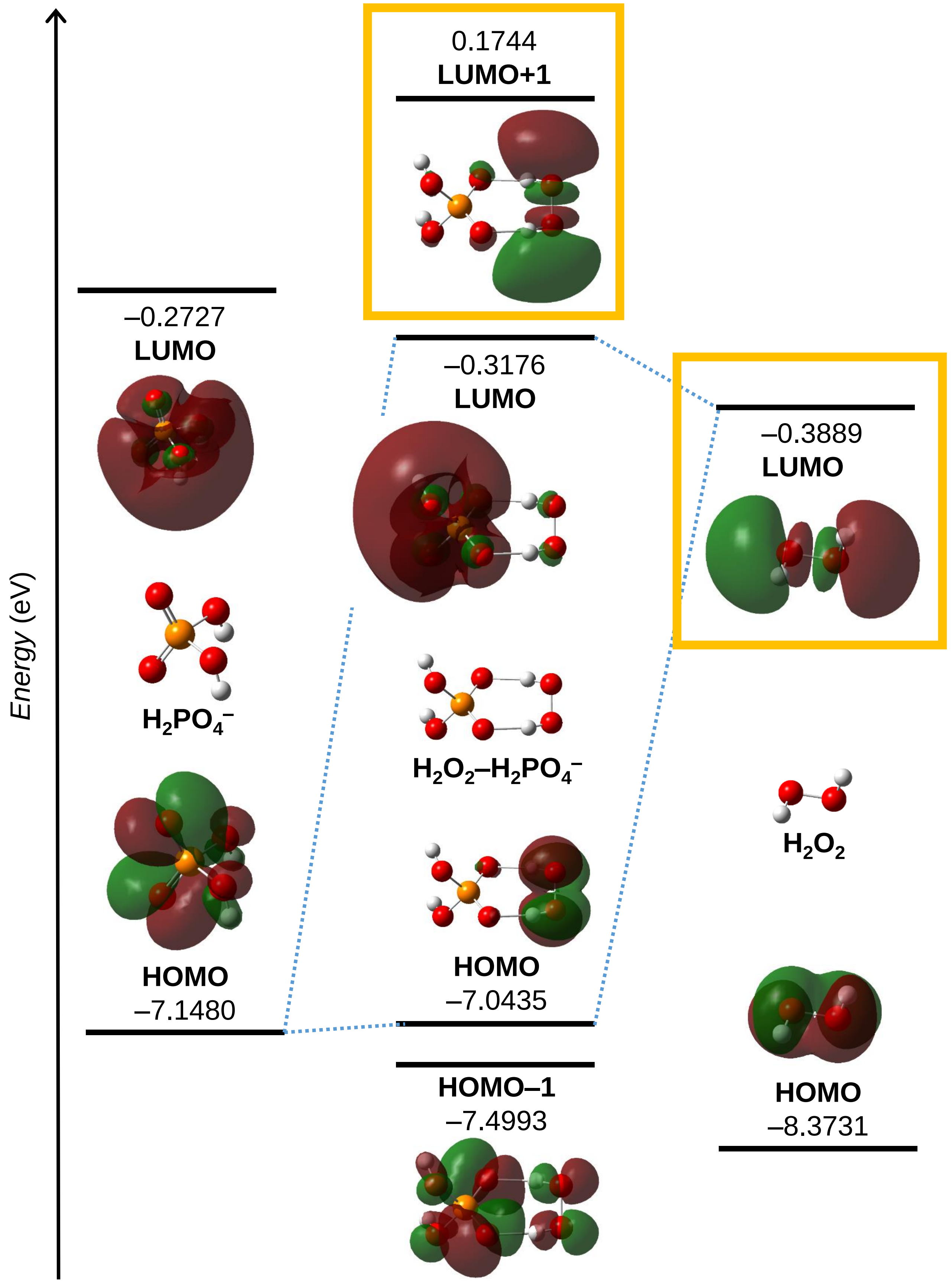


**Supplementary Fig. 5 | Energy diagrams and interfacial plots of the frontier orbitals for the calculated models (DFT/B3LYP/6–31G*(d), PCM: water).**

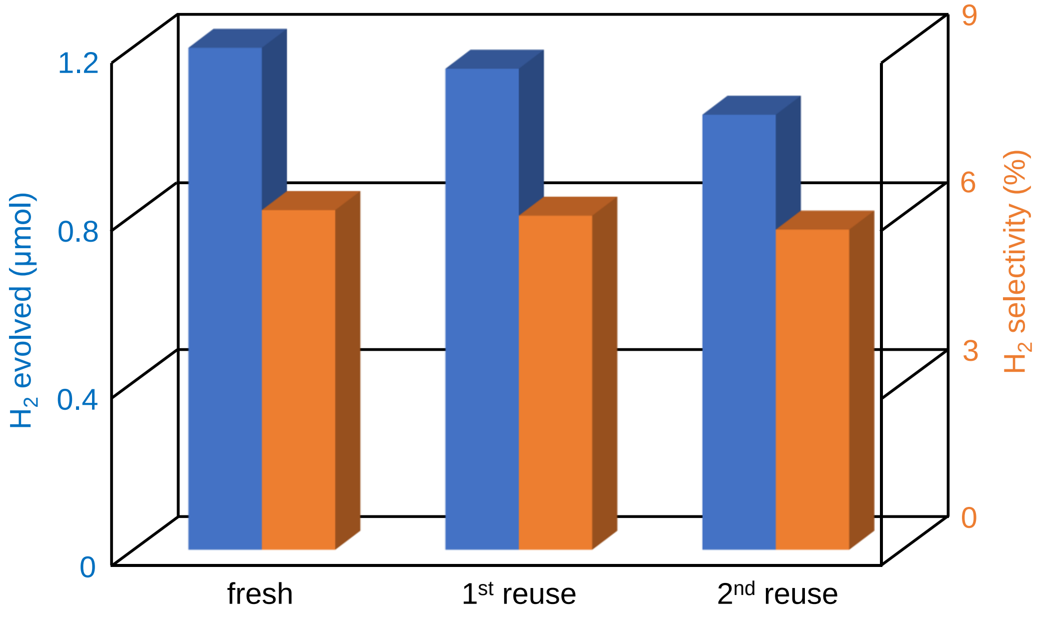


**Supplementary Fig. 6 | Results for repeated photocatalytic reactions with $GQD_1$/g-$C_3N_4$ under visible light irradiation.** The reactions were performed in a closed gas circulation system (3 kPa Ar). Conditions: catalyst (200 mg), $H_2O_2$ (10 mM, 100 mL), $H_3PO_4$ (1 M), temperature (293 K), light irradiation ($\lambda$ >420 nm, solar simulator with AM 1.5G filter, 1-sun, 6 h).

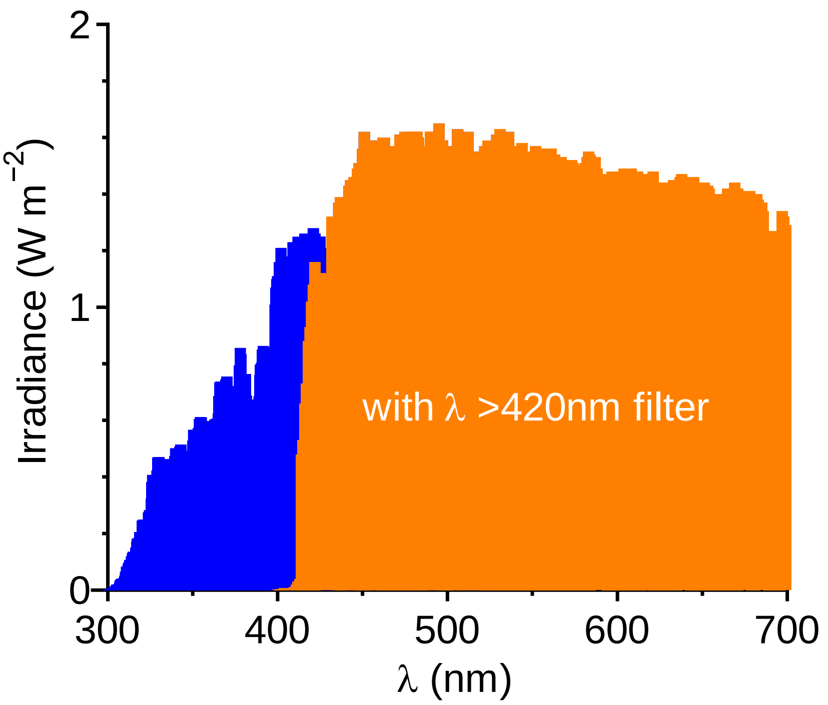


**Supplementary Fig. 7 | Light emission spectra of the simulated AM1.5G sunlight (1-sun) with or without λ >420 nm filter.**

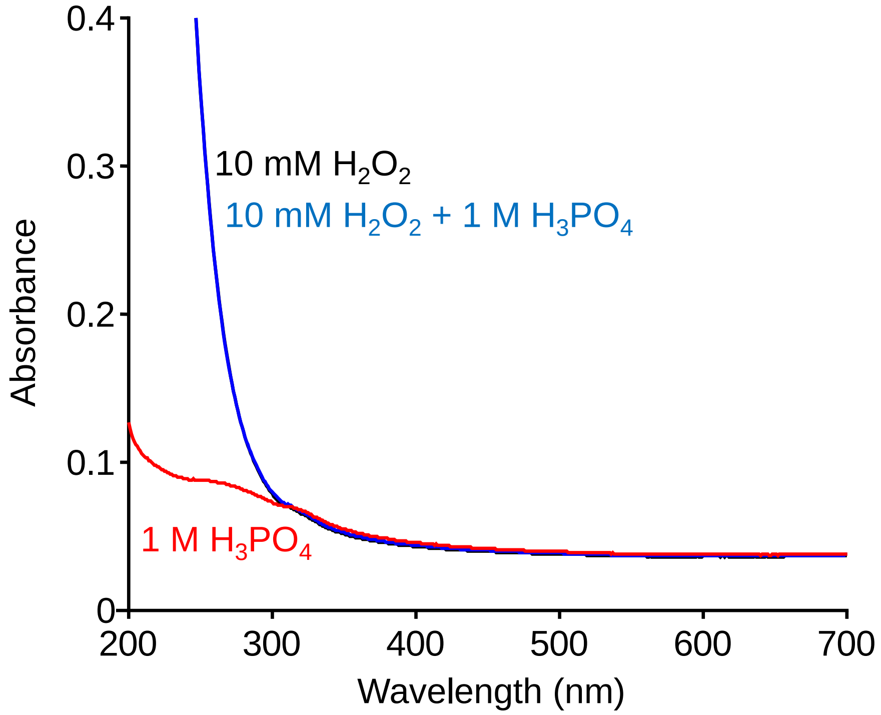


**Supplementary Fig. 8 | Absorption spectra of the respective solutions.**

**Supplementary Table 1 | Results of photocatalytic $H_2O_2$ splitting on $GQD_1$/g-$C_3N_4$ with the respective $H_2O_2$ stabilizers.**

| entry | stabilizer | references | concentration (M) | $H_2$ (μmol) | $O_2$ (μmol) | consumed $H_2O_2$ (μmol) |
|---|---|---|---|---|---|---|
| 1 | $H_3PO_4$ | | 1 | 1.20 | 7.7 | 20 |
| 2 | $H_3PO_3$ | [1] | 1 | N.D. | 3.1 | 10 |
| 3 | uric acid | [2] | 0.001[a] | N.D. | 3.6 | 12 |
| 4 | $Na_2CO_3$ | [3] | 1 | N.D. | 46.0 | 334 |
| 5 | $KHCO_3$ | [4] | 1 | N.D. | 11.9 | 77 |
| 6 | barbituric acid | [5] | 0.01[a] | 0.14 | 5.1 | 11 |
| 7 | hippuric acid | [6] | 0.01[a] | N.D. | 5.0 | 11 |
| 8 | urea | [7] | 1 | N.D. | 9.9 | 28 |
| 9 | acetanilide | [7] | 0.01[a] | N.D. | 2.9 | 8 |

Reaction conditions: $GQD_1$/g-$C_3N_4$ catalyst (200 mg), $H_2O_2$ solution (1 mmol (10 mM), 100 mL), visible light (λ >420 nm, solar simulator, 1-sun), temperature (293 K), photoirradiation time (6 h). Reactions were performed in a closed gas circulation system (3 kPa Ar).

[a] Maximum solubility of the stabilizer in the solutions.

**Supplementary Table 2 | Calculated vibrational frequencies of the respective molecular models (DFT/B3LYP/6–31G*(d), PCM: water).**

| $H_2O_2$ $\tilde{\nu}$ ($cm^{-1}$) | $H_3PO_4$ $\tilde{\nu}$ ($cm^{-1}$) | $H_2O_2$–$H_2PO_4^-$ $\tilde{\nu}$ ($cm^{-1}$) | assignment |
|---|---|---|---|
| | | 23.9212 | |
| | | 102.3312 | |
| | | 150.0782 | |
| | | 204.8058 | |
| | | 232.2375 | |
| | 290.7909 | 323.5251 | |
| | 329.5801 | 342.7774 | |
| | 424.5292 | 453.8269 | |
| | 448.7212 | 470.4110 | |
| | | 480.7858 | |
| | | 557.3817 | O–P–O bending[8] |
| | | 604.0560 | |
| | 794.6220 | 712.9356 | |
| | 810.8800 | 802.8116 | P–OH symmetric stretching[9] |
| | 869.2789 | 834.1122 | P–OH asymmetric stretching[9] |
| 947.6856 | | 942.7756 | O–O stretching[10] |
| | 1074.8559 | 1038.8478 | P–O symmetric stretching[11] |
| | 1235.0546 | 1210.9805 | P–O asymmetric stretching[12] |
| 1408.7728 | 1359.1172 | 1498.4483 | O–H asymmetric bending[10] |
| 1460.9403 | | 1541.9975 | O–H symmetric bending[10] |
| 3456.7391 | | 3245.9582 | |
| 3460.8525 | | 3304.3653 | |
| | | 3481.0303 | |
| | | 3486.1007 | |
| | 4135.0326 | | |
| | 4154.3765 | | |
| | 4160.2412 | | |

**Supplementary Table 3 | Cartesian Coordinates (in Å) of $H_2O_2$**

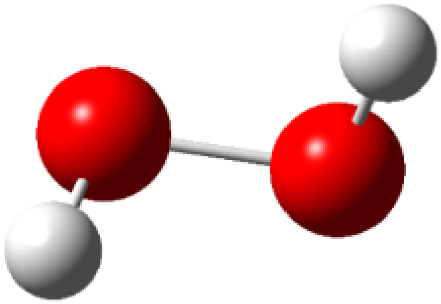

| | | | |
|---|---|---|---|
| O | -0.7193 | 0.108806 | -0.06825 |
| O | 0.719301 | -0.10881 | -0.06824 |
| H | -1.02936 | -0.60826 | 0.545958 |
| H | 1.029321 | 0.608318 | 0.545916 |

**Supplementary Table 4 | Cartesian Coordinates (in Å) of $H_3PO_4$**

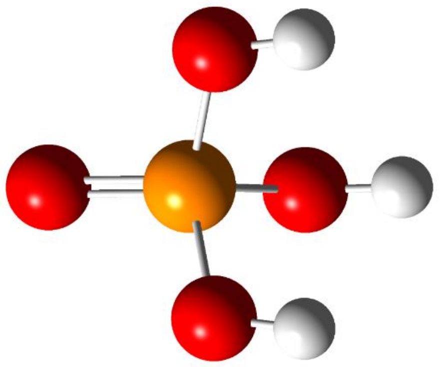

| | | | |
|---|---|---|---|
| O | 1.620213 | -0.042263 | 0.029374 |
| P | 0.134336 | -0.003222 | 0.002435 |
| O | -0.447534 | -0.931011 | -1.174587 |
| O | -0.484173 | -0.525314 | 1.391143 |
| O | -0.400132 | 1.493042 | -0.242805 |
| H | -1.482641 | -0.519154 | 1.445052 |
| H | -1.394578 | 1.590304 | -0.278671 |
| H | -1.444811 | -0.978447 | -1.227897 |

**Supplementary Table 5 | Cartesian Coordinates (in Å) of $H_2PO_4^-$**

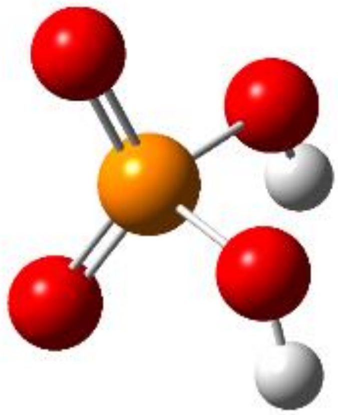

| | | | |
|---|---|---|---|
| O | -0.08398 | -0.000091 | -0.05146 |
| P | 0.049083 | -0.000018 | 1.45529 |
| O | 1.411997 | 0.000364 | 2.126045 |
| O | -0.82121 | 1.297371 | 1.988653 |
| O | -0.82095 | -1.2976 | 1.988474 |
| H | -0.65428 | 1.479286 | 2.948008 |
| H | -0.65645 | -1.47775 | 2.948607 |

**Supplementary Table 6 | Cartesian Coordinates (in Å) of $H_2O_2$–$H_2PO_4^-$ complex**

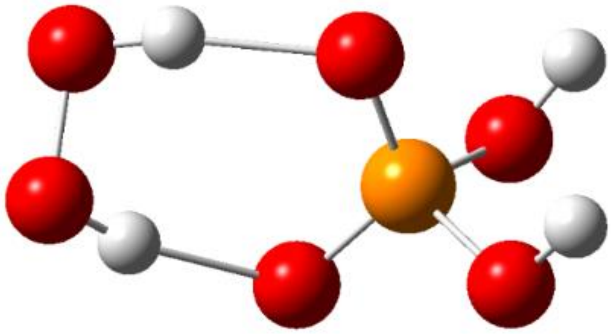

| | | | |
|---|---|---|---|
| O | 4.156013 | 0.724329 | 0.001358 |
| O | 4.219231 | -0.74244 | -0.00144 |
| H | 5.126953 | 0.939431 | -0.01973 |
| H | 3.258924 | -0.96827 | 0.01358 |
| O | -0.17355 | 0.791371 | 0.009606 |
| P | -1.55689 | -0.12078 | 0.001075 |
| O | -2.62121 | 1.143483 | -0.05284 |
| O | -1.64105 | -0.93573 | -1.27919 |
| O | -1.70133 | -0.86248 | 1.320817 |
| H | 0.586897 | 0.168834 | 0.045371 |
| H | -3.52428 | 0.723327 | -0.04187 |

## Supplementary References


[1] Pougherty, E. F. Stabilized Hydrogen Peroxide. US patent US4981662A (1991).

[2] Hooper, G. W. Catalytic Production of Hydrogen Peroxide from Its Elements. US patent US3336112A (1967).

[3] Lee, H. H. B., Park, A. H. & Oloman, C. Stability of Hydrogen Peroxide in Sodium Carbonate Solution. *TAPPI J. Peer Rev. Pap.* 1–9 (2000).

[4] Fuku, K., Miyase, Y., Miseki, Y., Funaki, T., Gunji, T. & Sayama, K. Photoelectrochemical Hydrogen Peroxide Production from Water on a $WO_3$/$BiVO_4$ Photoanode and from $O_2$ on an Au Cathode Without External Bias. *Chem. Asian J.* **12**, 1111–1119 (2017).

[5] Malin, M. J. & Sclafani, L. D. Stabilized Aqueous Hydrogen Peroxide Solution. US patent US4744968A. (1988).

[6] Weber, F. W. Stable Product Containing Hydrogen Peroxide and Method of Making the Same. US patent US1210570A (1917).

[7] Baum, G. Stabilized Peroxide Solution. US patent US1758920A (1930).

[8] Yaguchi, M., Uchida, T., Motobayashi, K. & Osawa, M. Speciation of Adsorbed Phosphate at Gold Electrodes: A Combined Surface-Enhanced Infrared Absorption Spectroscopy and DFT Study. J. Phys. *Chem. Lett.* **7**, 3097–3102 (2016).

[9] VandeVondele, J., Tröster, P., Tavan, P. & Mathias, G. Vibrational Spectra of Phosphate Ions in Aqueous Solution Probed by First Principles Molecular Dynamics. *J. Phys. Chem. A* **116**, 2466–2474 (2012).

[10] Gonzalez, L., Mo, O. & Yanez, M. High-level Ab Initio Versus DFT Calculations on $(H_2O_2)_2$ and $H_2O_2$–$H_2O$ complexes as Prototypes of Multiple Hydrogen Bond Systems. *J. Comput. Chem.* **18**, 1124–1135 (1997).

[11] Ahmed, A. B., Feki, H., Abid, Y., Boughzala, H., Minot, C. & Mlayah, A. Crystal Structure, Vibrational Spectra and Theoretical Studies of L-Histidinium Dihydrogen Phosphate-Phosphoric Acid. *J. Mol. Struct.* **920**, 1–7 (2009).

[12] Rudolph, W. W. Raman- and Infrared-Spectroscopic Investigations of Dilute Aqueous Phosphoric Acid Solutions. *Dalton Trans.* **39**, 9642–9653 (2010).
