# Supplementary material for: Photocatalytic hydrogen peroxide splitting on metal-free powders assisted by phosphoric acid as a stabilizer
Source: Nat Commun. 2020 Jul 7;11:3386. doi: 10.1038/s41467-020-17216-2 (PMC7341847; doi:10.1038/s41467-020-17216-2)
Supplement: Supplementary file 2 — Peer Review File [file 41467_2020_17216_MOESM2_ESM.docx]

Peer Review Information

**Journal:** Nature Communications

**Manuscript title:** Photocatalytic hydrogen peroxide splitting on metal-free powders assisted by phosphoric acid as a stabilizer

**Corresponding author name(s):** Dr Yasuhiro Shiraishi

**Editorial notes:**

none

**Reviewer comments & decisions:**

**Reviewer comments, first version:**

Reviewer #1 (Remarks to the Author):

This is an interesting paper. It seems that this reaction has not been reported for photocatalytic assisted reactions. There are some issues to address though. P1, for all equations, give all states of matter. take out we find that. The abstract should quantitatively summarize the findings of the work. P2, the equations should have all states of matter. P3, Equations 3-9 give all states. define the word It. take out we found that. P5, take out it is noted that. take out this indicates that. P6, take out this suggests that. take out this suggests that. take out this is because. take out to the best of our knowledge. take out this indicates that. P7, define larger. Take out this again suggests that. take out it is generally considered that. define larger. P8, take out These data clearly indicate that. Take out this implies that. P9, take out it is noted that. take out these finding clearly suggest that. P10, take out these findings indicate that. P12, take out we found that. Figures 1b,d - give error bars on all data points. Figure 2, explain the red arrows in the Figure 2 caption. Figure 4, what is the peak at about 1650 cm-1?


Reviewer #2 (Remarks to the Author):

In this manuscript, the authors reported on the photo-decomposition of H2O2 into H2 and O2, rather than the conventional H2O and O2, using GQDs loaded carbon nitrides. This results could be interesting from the chemistry viewpoint. However, I really cannot agree to regard H2O2 as an energy carrier although the authors have tried hard to experimentally produce H2 from it. It is a well-known fact that H2O2 is a very useful chemical in many areas, and in my opinion to develop new, greener and lower cost approach for H2O2 production would make much more sense.

Another serious concern is that the H2 was not determined to originate from H2O2; it may be formed by photocatalytic water splitting (since GQD/C3N4 is a very good photocatalyst) and even from the decomposition of TEOA agent.

Moreover, the authors have highlighted the advantages of using metal-free photocatalysts and visible light to drive the H2O2 reaction, however, using such a material and light source is so common in this field and I did not see it as a novelty.

Overall, I do not think the manuscript should be published in Nature Comm based on the above augment.


Reviewer #3 (Remarks to the Author):

The manuscript by Shiraishi et al. demonstrates photocatalytic hydrogen production by decomposition of hydrogen peroxide (H2O2), using a photocatalyst system composed of graphitic carbon nitride with graphene quantum dots as co-catalysts, with added phosphoric acid. Addition of phosphoric acid suppresses disproportionation of H2O2 and therefore enables the competing process of reduction of H+ to H2. This is a remarkable achievement, which may lead to a new convenient way to generate H2, by first oxidising water to H2O2, which then acts as the intermediate in producing H2. Phosphoric acid is demonstrated to be the key component necessary to achieve generation of H2 from hydrogen peroxide. Insights from Raman spectroscopy and quantum chemistry modelling are used to illustrate the nature of interaction between phosphoric acid and H2O2; this strong intermolecular interaction is believed to be responsible for suppression of H2O2 disproportionation and opens the way for reduction of H2O2 protons to H2. This is a very interesting result that is likely to inspire further research.
However, I have several comments (both technical: comments 1-2, and discussion of mechanisms: comments 3-4), that need addressing before publication:
1. The data in Table 1 on the amounts of H2 and O2 produced seem to show only one experiment for each entry (i.e. for each photocatalytic system and conditions). I recommend reporting mean values for several experiments done under identical conditions, and reporting their standard deviations.
2. The computational part of the work considers the interaction of H2O2 with the dihydrophosphate anion (H2PO4-). Why is the anion used rather than the neutral non-dissociated form of phosphoric acid (H3PO4)? At the very low pH used in these experiments (pH 1, p. 6-7), the non-dissociated form is likely to be predominant. I recommend calculating the equilibrium ratio of H3PO4 and H2PO4- at this pH, and repeating DFT calculations to describe the interaction of H2O2 with neutral H3PO4.
3. The manuscript does not offer an in-depth analysis of the mechanism whereby the H2O2-phosphoric acid interaction suppresses H2O2 disproportionation. It is demonstrated that such interaction exists (p. 9 and Fig. 5), and then it is simply stated “This suppresses the H2O2 reduction by the CB e–“, without explaining how this interaction may affect electro- and photochemical processes such as disproportionation.
4. The Raman spectroscopy data indicate that the H2O2–phosphoric acid complex is destabilized at high temperatures. What is the reason for this destabilisation? H2O2– H2PO4- electrostatic interaction is likely to be strong enough to survive at 313 K (e.g. hydrogen bonding in water survives until 373 K). This result also suggests that H3PO4 may be a better candidate for the species present in solution, because H2O2–H3PO4 interaction is likely to be weaker that for H2O2– H2PO4-. Calculated binding energies of these complexes would be useful here.

**Author rebuttal, first version:**

**Answers to comments by Reviewer #1**

(Comment 1) *There are some issues to address though.*

P1, *for all equations, give all states of matter. take out we find that. The abstract should quantitatively summarize the findings of the work.*
P2, *the equations should have all states of matter.*
P3, *Equations 3-9 give all states. define the word It. take out we found that.*
P5, *take out it is noted that. take out this indicates that.*
P6, *take out this suggests that. take out this suggests that. take out this is because. take out to the best of our knowledge. take out this indicates that.*
P7, *define larger. Take out this again suggests that. take out it is generally considered that. define larger.*
P8, *take out These data clearly indicate that. Take out this implies that.*
P9, *take out it is noted that. take out these finding clearly suggest that.*
P10, *take out these findings indicate that.
     P12, take out we found that.*

Parts of the manuscript were revised according to the suggestion. New descriptions about the quantitative results of the present photocatalytic system were made in the abstract.

(Comment 2) *Figures 1b,d - give error bars on all data points.*

The error bars were added to these figures.

(Comment 3) *Figure 2, explain the red arrows in the Figure 2 caption.*

The red arrows are the guide for the eyes to follow the GQD particles. To clarify this, new descriptions were added to the caption.

(Comment 4) *Figure 4, what is the peak at about 1650 cm-1?*

The Raman shifts observed at ~1650 cm^–1^ for all samples are assigned to O–H bending of water molecules (ref 52). To clarify this, its assignment was added to Fig. 4a.

1

**Answers to comments by Reviewer #2**

(Comment 1) *In this manuscript, the authors reported on the photo-decomposition of H2O2 into H2 and O2, rather than the conventional H2O and O2, using GQDs loaded carbon nitrides. This results could be interesting from the chemistry viewpoint. However, I really cannot agree to regard H2O2 as an energy carrier although the authors have tried hard to experimentally produce H2 from it. It is a well-known fact that H2O2 is a very useful chemical in many areas, and in my opinion to develop new, greener and lower cost approach for H2O2 production would make much more sense.*

As described in the introduction part, H2O2 has received increasing attention as a new energy carrier because it is a storable and transportable liquid and can be used as a fuel for electricity generation. A recent review article (ref 8) summarizes its potential activity as a fuel. The most attractive feature is that H2O2 can be generated from water and O2 by photocatalysis (equation (2)). The photocatalytic H2O2 generation is therefore considered as a new promising process for solar fuel production. Recent rapid progress in this field have demonstrated that some metal-free catalysts successfully produce H2O2 with 0.5% of solar-to-chemical conversion efficiency, which is the highest efficiency achieved by powder photocatalysts in artificial photosynthesis reactions. Considering such circumstance, the possibility of H2O2 as an energy carrier is expected to increase further in the near future. For the practical use of H2O2, on-site generation of H2 from H2O2 by photocatalysis is an essential technology (equation (5)), although the reaction has not been achieved due to the several problems. In the present work, we report the first successful example of this reaction by solving these problems. Although further improvement of the system is necessary, the results could contribute to inspire further research on photocatalytic H2O2 splitting. We believe that the ideas and discussions in this work provide important information to the readers. To clarify the above, the introduction part was revised (page 2).

(Comment 2) *Another serious concern is that the H2 was not determined to originate from H2O2; it may be formed by photocatalytic water splitting (since GQD/C3N4 is a very good photocatalyst) and even from the decomposition of TEOA agent.*

As shown by new entry 3 (Table 1), photoreaction of the GQD1/g-C3N4 catalyst in pure water does not produce H2 or O2. This is because g-C3N4 is less active for water oxidation by the VB h+ due to its relatively negative VB level, as reported in several literatures (for example: ref 35). This clearly indicates that H2O2 is the electron donor for H2 generation. To clarify the above, new descriptions were made (page 6).

(Comment 3) *Moreover, the authors have highlighted the advantages of using metal-free photocatalysts and visible light to drive the H2O2 reaction, however, using such a material and light source is so common in this field and I did not see it as a novelty.*

As described in the manuscript, several metal-free catalysts related to GQD and g-C3N4 have already been reported. However, the important point of this work is ***the simultaneous use of metal-free catalyst and H3PO4 under visible light***. This novel idea facilitates photocatalytic H2O2 splitting for the first time. We believe that the findings of this study are relevant to the broad scope of Nature Communications and will be of interest to its readership. To clarify the above, the descriptions were revised (page 12).

2

**Answers to comments by Reviewer #3**

(Comment 1) *The data in Table 1 on the amounts of H2 and O2 produced seem to show only one experiment for each entry (i.e. for each photocatalytic system and conditions). I recommend reporting mean values for several experiments done under identical conditions, and reporting their standard deviations.*

The reactions in Table 1 were performed multiple times under identical conditions to confirm the reproducibility of the data. Therefore, the values in this table are the mean values obtained by these experiments. The experimental results indicated that all of the data (amounts of H2 and O2 formed and H2O2 consumed) contain less than 21% deviations. To clarify this, new descriptions were made in the footnote of Table 1.

(Comment 2) *The computational part of the work considers the interaction of H2O2 with the dihydrophosphate anion (H2PO4-). Why is the anion used rather than the neutral non-dissociated form of phosphoric acid (H3PO4)? At the very low pH used in these experiments (pH 1, p. 6-7), the non-dissociated form is likely to be predominant. I recommend calculating the equilibrium ratio of H3PO4 and H2PO4- at this pH, and repeating DFT calculations to describe the interaction of H2O2 with neutral H3PO4.*

As suggested, phosphoric acid exists as a neutral non-dissociated H3PO4 form in a low pH solution. Based on ref 50, the mole ratio of the H3PO4 form at pH ~1 is ~94%, whereas the mono-protonated H2PO4^–^ form is only ~6%. This means that H2O2 interacts with H3PO4 in the solution. To clarify this, new descriptions were made (page 8, top). According to this revision, DFT calculations were performed using the H3PO4 model (Fig. 5b (middle)). The frequency calculation data (Supplementary Table 2) were replaced with new data, and the H-bonding model (Fig. 5a) were revised referring with the ref 50. The related descriptions in the manuscript were revised (page 8–9).

(Comment 3) *The manuscript does not offer an in-depth analysis of the mechanism whereby the H2O2-phosphoric acid interaction suppresses H2O2 disproportionation. It is demonstrated that such interaction exists (p. 9 and Fig. 5), and then it is simply stated “This suppresses the H2O2 reduction by the CB e–“, without explaining how this interaction may affect electro- and photochemical processes such as disproportionation.*

New Supplementary Fig. 5 shows the energy diagram for the frontier orbitals of the calculated models. The interaction between the HOMO of H2PO4^–^ and the LUMO of H2O2 creates a frontier orbital of the complex (LUMO+1) at an energy level higher than that of free H2O2. This clearly means that the H2O2 moiety of the complex is stabilized against its reduction upon external electron injection. This is because the electron donation from H2PO4^–^ to H2O2 leads to a decrease in the electron affinity of the H2O2 moiety. The result is consistent with the suppressed H2O2 reduction (Fig. 3) and the enhanced H2 evolution (Table 1, entry 6) by the addition of H3PO4. Therefore, H3PO4 associates with H2O2 via H-bonding and produces a stabilized complex (Fig. 5a (right) and Fig. 5b (right)). This interaction decreases the electron affinity of H2O2 and suppresses the H2O2 reduction by the CB e^–^ (equations (8) and (9)), thus resulting in the promotion of H^+^ reduction (equation (4)). To clarify this, new descriptions were made (page 9 bottom–page 10 top) referring with new refs 60 and 61.

3

(Comment 4) *The Raman spectroscopy data indicate that the H2O2–phosphoric acid complex is destabilized at high temperatures. What is the reason for this destabilisation? H2O2– H2PO4-electrostatic interaction is likely to be strong enough to survive at 313 K (e.g. hydrogen bonding in water survives until 373 K). This result also suggests that H3PO4 may be a better candidate for the species present in solution, because H2O2–H3PO4 interaction is likely to be weaker that for H2O2– H2PO4-. Calculated binding energies of these complexes would be useful here.*

The H2O2–H2PO4– complex is destabilized at high temperatures because a rise in temperature increases the kinetic energy of the molecules and weakens the H-bonding interaction, as observed in general H-bonding systems such as DMSO–water and urea–water at a similar temperature range (283–333 K). The strong H-bonding between “water” molecules (~373 K) originates from their high molecular density. To clarify the above, new descriptions were made (page 10 bottom) referring with new refs 62–64.

As shown in Fig. 5a (right) and answered to the comment (2), H3PO4 interacts with H2O2 and produces **the H2O2–H2PO4^–^ complex** via a deprotonation, where **the H2O2–H3PO4 complex as you suggested may not form**. DFT calculation of the H2O2–H3PO4 complex provides the optimized structure shown below (right). Note that, during the structural optimization, only this initial structure converged the calculation, meaning that **the H2O2–H3PO4 complex is significantly unstable**. The O–O bond length in this complex (1.455 Å) is identical to that in free H2O2 (1.455 Å, Fig. 5b). In addition, the average P–O length in this complex (1.576 Å) is shorter than that of free H3PO4 (1.578 Å). These results are inconsistent with the lower wavenumber shift of the O–O and P–O stretching vibrations observed by the Raman analysis (Fig. 6a). This suggests that the H2O2–H3PO4 complex may not form in the present system, leaving the H2O2–H2PO4– complex as the possible species in this system. In addition, the structure of the H2O2–H2PO4– complex is very similar to the urea–H2PO4^–^ 1:1 bidentate complex involving two H‧‧‧O‧‧‧P moieties (ref 51). This strongly supports the H2O2–H2PO4– complex. To clarify this, new descriptions were made (page 8 middle). Note that we did not make descriptions on the above DFT calculations of the H2O2–H3PO4 complex, because the convergence condition is very limited and the descriptions if we made may lead to a confusion and misunderstanding of the readers.

H2O2–H3PO4 complex

1.46210

1.52541

P

O5

1.45503

H1	1.77518

O1	O3

H3

H1

1.82263

O3

1.60023

H3

0.99693

O1

0.98901

1.49032

O5

1.60688

1.75891

O4

O6

0.99798

P

1.60659

H4

O2

0.97856

H2

1.63724

O6

H4

O4

H5

H2O2–H2PO4– complex

O2

H2

1.51923

1.63698

2.20287

4

**Itemized list of changes**

**To Reviewer #1**

(Comment 1) Parts of the manuscript were revised. New descriptions were made in the abstract.

(Comment 2) Fig. 1b and d were revised.

(Comment 3) New description was added to the caption of Fig. 2.

(Comment 4) Fig. 4a was revised.

**To Reviewer #2**

(Comment 1) The descriptions were revised (page 2) referring with new refs 10, 11, and 13.

(Comment 2) New data (entry 3) was added to Table 1. New descriptions were made (page 6)

referring with new ref 35.

(Comment 3) The descriptions were revised (page 12).

**To Reviewer #3**

(Comment 1) New descriptions were made in the footnote of Table 1. Some of the data in Table 1

were revised by the additional experiments.

(Comment 2) Parts of the manuscript were revised (page 8–9). Fig. 5a, 5b, and Supplementary

Table 2 were revised.

(Comment 3) New Supplementary Fig. 5 was added. New descriptions were made (page 9 bottom–

page 10 top) referring with new refs 60 and 61.

(Comment 4) New descriptions were made (page 8 middle; page 10 bottom) referring with new

refs 51 and 62–64.

**Reviewer comments, second version:**

Reviewer #2 (Remarks to the Author):

In the rebuttal letter, the authors tried to highlight the significance and novelty of the submitted work. However, this reviewer was still not convinced based on the below concerns:

1. The authors stated that H2O2 is an emerging energy carrier and experimentally produced H2 from H2O2 via photocatalysis. However, it is common knowledge that H2O2 is thermodynamically unstable and easily decompose into OXGYEN and water by mild heating, UV lighting or using very simple catalysts (e.g. Fe3+, enzyme). In this sense, it make much more sense to produce O2 rather than H2 from H2O2.
2. Following up, the decomposition of H2O2 release a lot of heat in addition to oxygen, which can be dangerous may cause immediate fire! It is not feasible to use H2O2 as an energy carrier, especially with H2O being extensively studied as H2 source.
3. Several chemical reaction equations were provided to endorse the authors' argument. However, simply writing up reaction equations did not justify the practical value, significance and feasibility of the reported approach of producing H2 from H2O2.
4. As this reviewer already pointed out, H2O2 itself is a highly valuable and extremely useful chemicals for bleaching, producing organic compounds, and used as propellant and disinfectant, and many others. In this context, it did not make sense to produce H2 from H2O2; but on the contrary, the authors should try more to produce H2O2 from water.
5. What is the rational of using H3PO4 to facilitate the photoactivity? did the authors just accidently pick this one? how about other acid and salts, such as HCl, H2SO4, Na3PO4, Na2HPO4 and NaH2PO4?

Overall, this reviewer believes although something new was provided in the manuscript, there are not enough scientific insights and meaningful applications. This contribution does not meet the standards of Nature Communications.


Reviewer #3 (Remarks to the Author):

I am sufficiently satisfied with the authors' response. The manuscript can now be published.

**Author rebuttal, second version:**

**Answers to comments by Reviewer #2**

(Comment 1) *The authors stated that H2O2 is an emerging energy carrier and experimentally produced H2 from H2O2 via photocatalysis. However, it is common knowledge that H2O2 is thermodynamically unstable and easily decompose into OXGYEN and water by mild heating, UV lighting or using very simple catalysts (e.g. Fe3+, enzyme). In this sense, it make much more sense to produce O2 rather than H2 from H2O2.*

To realize a sustainable energy society with H_2_O_2_, on-site generation of H_2_ from a H_2_O_2_ solution is necessary for its use as a “hydrogen” carrier. However, the photocatalytic H_2_O_2_ splitting (equation (5)) is very difficult to promote because the decomposition of H_2_O_2_ into water and O_2_ (equation (6)) occurs thermodynamically more favorably. We therefore studied the way for promoting the H_2_O_2_ splitting reaction. To clarify the above, some texts were revised (page 2 bottom).

H_2_O_2_ (l) $\underset{\to}{hv}$ H_2_ (g) + O_2_ (g) (Δ*G*° = +131 kJ mol^–1^)	(5)

H_2_O_2_ (l) → H_2_O (l) + 1/2O_2_ (g) (Δ*G*° = –117 kJ mol^–1^)	(6)

(Comment 2) *Following up, the decomposition of H2O2 release a lot of heat in addition to oxygen, which can be dangerous may cause immediate fire! It is not feasible to use H2O2 as an energy carrier, especially with H2O being extensively studied as H2 source.*

It is well known that H_2_O_2_ has the property of being decomposed in the presence of some metal impurities or under heating conditions. However, the production amount of H_2_O_2_ is so large that its storage and transportation methods have been well established. H_2_O_2_ can therefore be a potential energy carrier. To clarify the above, some descriptions were made (page 2 top).

(Comment 3) *Several chemical reaction equations were provided to endorse the authors' argument. However, simply writing up reaction equations did not justify the practical value, significance and feasibility of the reported approach of producing H2 from H2O2.*

The significance of photocatalytic H_2_O_2_ splitting has already been described in the manuscript. The present work reported that this reaction can be achieved by an all-organic catalyst with H_3_PO_4_ as a stabilizer, clearly demonstrating the feasibility of this approach. However, at present, H_2_ selectivity is only ~6% relative to the amount of H_2_O_2_ consumed, and further improvement of its selectivity is necessary for practical applications.

(Comment 4) *As this reviewer already pointed out, H2O2 itself is a highly valuable and extremely useful chemicals for bleaching, producing organic compounds, and used as propellant and disinfectant, and many others. In this context, it did not make sense to produce H2 from H2O2; but on the contrary, the authors should try more to produce H2O2 from water.*

As already described in the manuscript, several photocatalytic systems for H_2_O_2_ production from water and O_2_ have been proposed. In particular, recently, we reported that some metal-free powder catalysts successfully produce H_2_O_2_ with 0.5% of solar-to-chemical conversion efficiency, which is the highest efficiency achieved by powder photocatalysts in artificial photosynthesis reactions (ref 13). It is well known that H_2_O_2_ can be used as a fuel for electricity generation. These circumstances enhance the applicability of H_2_O_2_ as an “energy” carrier. To realize a sustainable energy society with H_2_O_2_, on-site generation of H_2_ from a H_2_O_2_ solution is necessary for the use as a “hydrogen” carrier. Therefore, in the present work, we studied the feasibility of photocatalytic H_2_O_2_ splitting.

(Comment 5) *What is the rational of using H3PO4 to facilitate the photoactivity? did the authors just accidently pick this one? how about other acid and salts, such as HCl, H2SO4, Na3PO4, Na2HPO4 and NaH2PO4?*

As described in the manuscript, H_3_PO_4_ is used as a stabilizer for commercially available H_2_O_2_ solution. We therefore used it for the photoreactions. As shown in Table 1 and Supplementary Table 1, the photoreaction results obtained using several acids revealed that phosphoric acid specifically enhances H_2_ evolution as a stabilizer. It must be noted that addition of H_3_PO_4_ is necessary for efficient stabilization; addition of phosphate salts is ineffective. As shown by new entry 10 (Table 1), a NaH_2_PO_4_ (1 M) solution (pH ~1), when used for photoreaction, produces a lower amount of H_2_ than the H_3_PO_4_ (1 M, pH ~1) solution (entry 6) although fully protonated H_3_PO_4_ species exist mainly in both solutions. As reported (ref 62), alkaline metal cations interact with H_2_O_2_ in solution. The H_2_O_2_–Na^+^ interaction may weaken the H_2_O_2_–H_2_PO_4_^–^ complex, and results in the decreased H_2_ evolution activity. To clarify the above, new descriptions were made (page 10) referring with new ref 62.

**Reviewer comments, third version:**

**Author rebuttal, third version:**
